# The *E. coli* sirtuin CobB shows no preference for enzymatic and nonenzymatic lysine acetylation substrate sites

**DOI:** 10.1002/mbo3.223

**Published:** 2014-11-22

**Authors:** Alaa AbouElfetouh, Misty L Kuhn, Linda I Hu, Michael D Scholle, Dylan J Sorensen, Alexandria K Sahu, Dörte Becher, Haike Antelmann, Milan Mrksich, Wayne F Anderson, Bradford W Gibson, Birgit Schilling, Alan J Wolfe

**Affiliations:** 1 Department of Microbiology and Immunology, Loyola University Chicago, Health Sciences Division, Stritch School of MedicineMaywood, Illinois, 60153; 2 Department of Microbiology, Faculty of Pharmacy, Alexandria UniversityAlexandria, 21521, Egypt; 3 Department of Molecular Pharmacology and Biological Chemistry, Center for Structural Genomics of Infectious Diseases, Northwestern University Feinberg School of MedicineChicago, Illinois, 60611; 4 Departments of Biomedical Engineering Chemistry and Cell and Molecular Biology, Northwestern University2145 Sheridan Road, Evanston, Illinois, 60208; 5 Buck Institute for Research on AgingNovato, California, 94945; 6 Institute for Microbiology, Ernst-Moritz-Arndt-University of GreifswaldF.-L.-Jahn-Str. 15, D-17487, Greifswald, Germany; 7 Department of Pharmaceutical Chemistry, University of CaliforniaSan Francisco, California, 94143

**Keywords:** Acetyl phosphate, bacteria, crystallography, deacetylase, mass spectrometry, posttranslational modification

## Abstract

*N*^*ε*^-lysine acetylation is an abundant posttranslational modification of thousands of proteins involved in diverse cellular processes. In the model bacterium *Escherichia coli*, the *ε*-amino group of a lysine residue can be acetylated either catalytically by acetyl-coenzyme A (acCoA) and lysine acetyltransferases, or nonenzymatically by acetyl phosphate (acP). It is well known that catalytic acCoA-dependent *N*^*ε*^-lysine acetylation can be reversed by deacetylases. Here, we provide genetic, mass spectrometric, structural and immunological evidence that CobB, a deacetylase of the sirtuin family of NAD^+^-dependent deacetylases, can reverse acetylation regardless of acetyl donor or acetylation mechanism. We analyzed 69 lysines on 51 proteins that we had previously detected as robustly, reproducibly, and significantly more acetylated in a *cobB* mutant than in its wild-type parent. Functional and pathway enrichment analyses supported the hypothesis that CobB regulates protein function in diverse and often essential cellular processes, most notably translation. Combined mass spectrometry, bioinformatics, and protein structural data provided evidence that the accessibility and three-dimensional microenvironment of the target acetyllysine help determine CobB specificity. Finally, we provide evidence that CobB is the predominate deacetylase in *E. coli*.

## Introduction

*N*^*ε*^-lysine acetylation in eukaryotes is emerging as an abundant posttranslational modification that influences function, structure, stability, and/or location of thousands of proteins involved in diverse cellular processes (Glozak and Seto 2007; Yang and Seto 2008a; Choudhary et al. 2009, 2014; Hebert et al. 2013; Rardin et al. 2013). Recent reports provide compelling evidence that *N*^*ε*^-acetylation is also an abundant posttranslational modification in bacteria (Zhang et al. 2009, 2013; Wang et al. 2010; Kim et al. 2013; Lee et al. 2013; Weinert et al. 2013; Wu et al. 2013; Kuhn et al. 2014).

**Figure 1:**
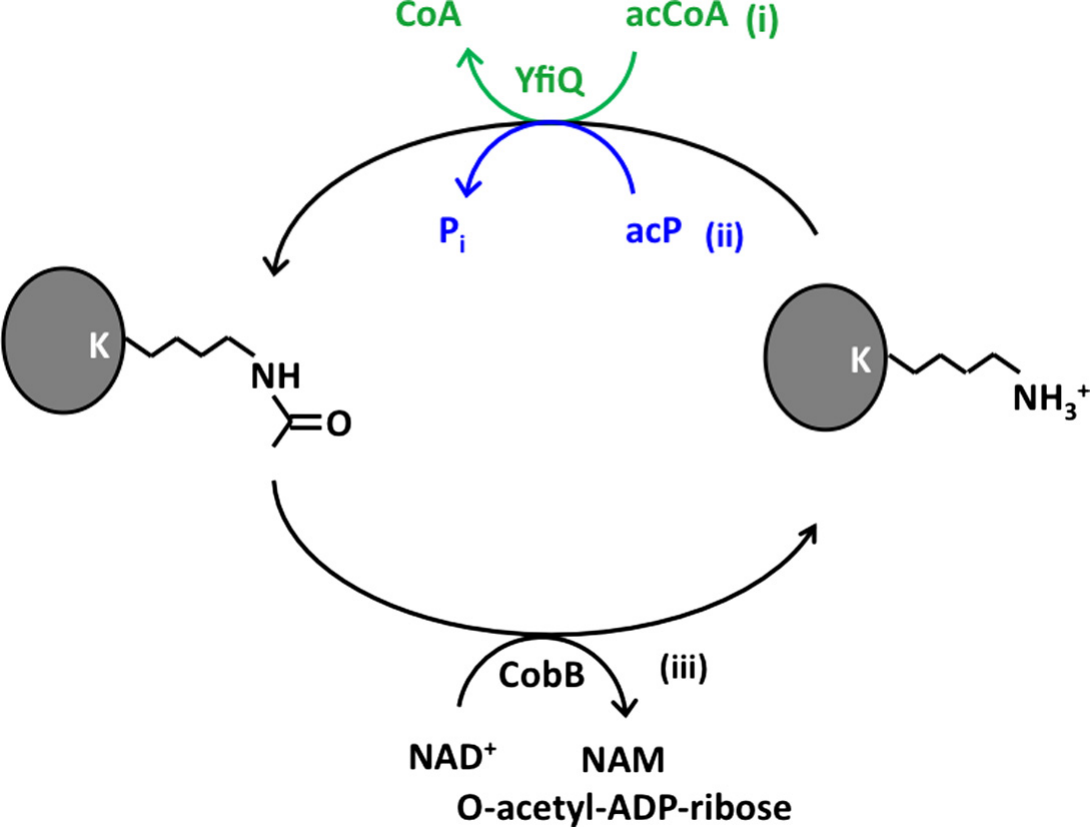
Schematic of *N*^*ε*^-lysine acetylation and deacetylation. K, lysine; P_i_, pyrophosphate; CoA, coenzyme A; acCoA, acetyl-coenzyme A; acP, acetyl phosphate, NAD^+^, nicotinamide adenine dinucleotide; NAM, nicotinamide. (i) The canonical enzymatic acetylation catalyzed by YfiQ and using acCoA as the acetyl donor (green); (ii) the nonenzymatic reaction using acP as the acetyl donor (blue); and (iii) CobB acting as deacetylase.

Two distinct mechanisms to acetylate bacterial proteins have been proposed. The first mechanism is enzymatic (Fig.1i), relying on a lysine acetyltransferase (KAT) to catalyze the donation of the acetyl group from acetyl-coenzyme A (acCoA) to the *ε*-amino group of a lysine residue (Hu et al. 2010; Soppa 2010; Jones and O'Connor 2011; Kim and Yang 2011; Thao and Escalante-Semerena 2011). In *Escherichia coli,* only one KAT has been identified. Known as YfiQ (also as Pat, PatZ, Pka, and Pla), this KAT belongs to the ubiquitous Gcn5-like family of acetyltransferases (GNATs) (Starai and Escalante-Semerena 2004). In contrast, the second mechanism is nonenzymatic (Fig.1ii). Acetyl phosphate (acP), the high-energy intermediate of the phosphotransacetylase (Pta) – acetate kinase (AckA) pathway (Wolfe 2005) directly donates its acetyl group to the deprotonated lysine *ε*-amino group (Weinert et al. 2013; Kuhn et al. 2014). The end result of both mechanisms is the same, acetylation of the *N*^*ε*^-amino group of a lysine residue within a protein. However, nonenzymatic acetylation with acP is more global and less specific than enzymatic acetylation (Weinert et al. 2013; Kuhn et al. 2014).

The resulting *N*^*ε*^-acetyllysine product is quite stable; however, it can be enzymatically reversed by lysine deacetylases (KDACs). Two KDAC families are known: a metal-dependent family (Yang and Seto 2008b) and the family of NAD^+^-dependent sirtuins (Blander and Guarente 2004). Putative bacterial homologs of each KDAC family have been identified (Hildmann et al. 2007), but few have been shown to serve as KDACs and few substrates have been reported (Starai et al. 2002; Gardner et al. 2006; Hildmann et al. 2007; Gardner and Escalante-Semerena 2009; Li et al. 2010; Wang et al. 2010). In *E. coli,* only one KDAC (the sirtuin CobB; Fig.1iii) has been reported (Starai et al. 2002; Hu et al. 2010; Thao and Escalante-Semerena 2011), and few CobB substrates have been identified (Starai et al. 2002; Thao et al. 2010). The best-studied CobB substrate is acCoA-synthetase (Acs), which synthesizes acCoA from acetate, ATP, and CoA (Wolfe 2005). The activity of this enzyme is inhibited by YfiQ-dependent acetylation (Starai and Escalante-Semerena 2004) and is reactivated by CobB-dependent deacetylation (Starai et al. 2002).

We recently confirmed that *N*^*ε*^-lysine acetylation is an abundant posttranslational modification in *E. coli* (Kuhn et al. 2014). We used a robust peptide-based affinity enrichment strategy with antiacetyllysine antibodies (Rardin et al. 2013) to detect 2730 unique acetylated lysine residues on 806 acetylated *E. coli* proteins that function in diverse and often essential cellular processes (Kuhn et al. 2014). We further used a novel, label-free quantitative mass spectrometric method called MS1 Skyline Filtering (Schilling et al. 2012; Rardin et al. 2013) to determine with statistical significance 592 lysines from 292 proteins that were sensitive to the levels of the novel acetyl donor acP. We also identified and quantified 69 lysines from 51 proteins that were sensitive to the deacetylase CobB (Kuhn et al. 2014).

With the discovery of thousands of newly identified acetyllysine sites (Weinert et al. 2013; Zhang et al. 2013; Kuhn et al. 2014), especially those sensitive to CobB (Kuhn et al. 2014), we deemed it imperative to address several pressing questions. What is the biological significance of the newly identified CobB substrates? Which biological pathways does CobB particularly target? By what mechanism does CobB find its specific deacetylation substrates? Does CobB discriminate between acetyl donors: does it deacetylate lysines acetylated by acP, as well as those acetylated by YfiQ?

To answer these questions, we performed bioinformatic analyses on the 51 CobB-sensitive proteins, learning that they are enriched primarily in translation, central metabolism, and DNA-centered processes. Using SAMDI (Self-Assembled monolayers with Matrix-assisted laser Desorption-Ionization) mass spectrometry, a label-free high-throughput peptide array technology (Gurard-Levin and Mrksich 2008; Mrksich 2008; Gurard-Levin et al. 2009), we obtained evidence that CobB is the predominate deacetylase in *E. coli*. SAMDI mass spectrometry also revealed a preference for certain amino acid residues in the positions adjacent to CobB-susceptible acetyllysines. Intriguingly, these characteristics did not match those identified in the linear (1D) sequences of the acetyllysines identified by our quantitative mass spectrometric analysis or by inspection of their three-dimensional (3D) environment, providing evidence that reliance on primary sequence alone to identify CobB targets is inadequate. With this in mind, we mapped the locations of the CobB-sensitive lysine residues onto previously determined protein structures in the Protein Data Bank (PDB), obtaining evidence that CobB-sensitive acetyllysines tend to be highly exposed lysines adjacent to alanine, glycine, tyrosine, and negatively charged resides. To determine whether CobB can deacetylate lysines acetylated by acP, we genetically altered the ability of cells to degrade acP, to deacetylate lysines, or to do both, and monitored global acetylation status by Western immunoblot analysis. This analysis revealed two classes of CobB-sensitive acP-dependent acetylations: those that are sensitive to CobB and those that are not, an assessment supported by quantitative mass spectrometric data and SAMDI analysis. Finally, we verified that CobB regulates YfiQ-dependent acetylation, providing support for the hypothesis that CobB can deacetylate acetyllysines whose acetyl groups were donated by either acCoA or acP.

## Methods

### Bacterial strains and culture conditions

All bacterial strains used in this study are listed in Table1. Derivatives were constructed by generalized transduction with P1kc, as described previously (Silhavy et al. 1984). For strain construction, cells were grown in LB containing 1% (w/v) tryptone, 0.5% (w/v) yeast extract, and 0.5% (w/v) sodium chloride; LB plates also contained 1.5% agar. Transformations were performed using transformation buffers 1 and 2 (Hanahan 1983). About 15 and 25 *μ*mol/L isopropyl *β*-d-1 thiogalactopyranoside (IPTG) were added to induce gene expression from plasmid vectors, unless otherwise mentioned. Tetracycline (15 *μ*g/mL), ampicillin (100 *μ*g/mL), and chloramphenicol (25 *μ*g/mL) were added to growth media when needed. For Western immunoblot analyses, cells were grown at 37°C in TB7 (1% [w/v] tryptone buffered at pH 7.0 with potassium phosphate [100 mmol/L]) or TB7 supplemented with 0.4% glucose. Cell growth was monitored spectrophotometrically (DU640; Beckman Instruments, Fullerton, CA) by determining the absorbance at 600 nm (*A*_600_).

**Table 1:** Strains and plasmids used in this study

	Relevant characteristic	Source/reference
Strain		
AJW678	*thi-1 thr-1*(Am) *leuB6 metF159*(Am) *rpsL136 ΔlacX74*	Kumari et al. (2000)
AJW1781	AJW678 *Δ(acsA::kan-1)*	Klein et al. (2007)
AJW2067	AJW678 *ackA*::Tn*phoA′*-2	Wolfe et al. (2003)
AJW2179	AJW678 *λ42(acs′-lacZ)*	Browning et al. (2004)
AJW3797	BW25113 *ΔyfiQ::frt kan*	Hu et al. (2013)
AJW4343	AJW678 *ΔcobB::frt kan λ42(acs′-lacZ)*	P1: JW1106→AJW2179
AJW4344	AJW678 *ΔyfiQ::frt kan λ42(acs′-lacZ)*	P1: JW2568→AJW2179
AJW4662	*ΔyfiQ::cm*	J. Escalante-Semerena (U of Georgia)
AJW5024	AJW678 *ΔackA*::Tn*phoA′*-2 *ΔcobB::frt kan*	P1: JW1106→AJW2067
AJW5109	AJW678 *ΔcobB::frt kan ΔyfiQ::cm*	P1: AJW4662→AJW4343
AJW5120	AJW678 *ΔcobB::frt λ42(acs′-lacZ)*	P1:JW1106→AJW2179 followed by antibiotic marker removal
AJW5121	AJW678 *ΔcobB::frt ΔyfiQ::frt kan λ42(acs′-lacZ)*	P1: AJW3797→AJW5120
AJW5185	AJW678 *ΔcobB::frt ΔacsA::kan λ42(acs′-lacZ)*	P1: AJW1781→AJW5120
MG1655	*λ- rph-1*	A. Ninfa (U of Michigan)
AJW5037	MG1655 *ΔcobB*::*cat*	P1: JE8659→MG1655
AJW5184	MG1655 *ΔyfiQ::frt kan*	P1:JW2568→MG1655
AJW5116	MG1655 *ΔcobB*::*cat ΔyfiQ::frt kan*	P1:JE8659→AJW5184
JW1106	*ΔcobB::frt kan*	Baba et al. (2006)
JW2568	*ΔyfiQ::frt kan*	Baba et al. (2006)
JE8659	*ΔcobB*::*cat*	J. Escalante-Semerena (U of Georgia)
Plasmid		
pCA24n	Control plasmid (CmR)	Kitagawa et al. (2005)
pCA24n-*yfiQ*	Plasmids expressing 6xHis-*yfiQ* under the control of an IPTG-inducible promoter (CmR)	Kitagawa et al. (2005)

### Functional analysis, protein ontology, pathway enrichment analysis

For functional analysis and protein ontology analysis “The Database for Annotation, Visualization and Integrated Discovery” (DAVID v.6.7) was used (Huang da et al. 2007). For pathway enrichment analysis, we used the Bonferroni correction as adjustment to *P*-values to determine statistical significance. To generate graphical ontology displays, Panther v8.1 classification system was used (Mi et al. 2013).

### Protein expression and purification of *E. coli* CobB

The CobB protein was produced from the ASKA collection clone [pCA24N, *E. coli* K-12 strain AG1 (Kitagawa et al. 2005)] and expressed and purified using procedures previously described for YfiQ and RcsB (Sanville et al. 2003; Kuhn et al. 2013, 2014).

### Peptide library synthesis, immobilization, assays, and SAMDI mass spectrometry

A peptide library composed of Ac-GXK(Ac)ZGC-CONH_2_, where X and Z represent all amino acids except cysteine, was synthesized and immobilized onto 384 SAMDI biochips and processed as described previously (Gurard-Levin et al. 2010, 2011; Kuhn et al. 2014). SAMDI biochip arrays with the immobilized Ac-GXK(Ac)ZGC-CONH_2_ peptide library or the immobilized Ac-GXKZGC-CONH_2_ peptide library treated with acP (Kuhn et al. 2014) were subjected to 100 nmol/L purified *E.coli* CobB in reaction buffer (25 mmol/L Tris-HCl pH 8.0, 137 mmol/L NaCl, 2.7 mmol/L KCl, 0.5 mmol/L dithiothreitol (DTT), 0.5 mmol/L NAD) for 30 min at 30**°**C. Negative controls were also performed, reactions were terminated, and biochips were analyzed with mass spectrometry using previously described procedures (Kuhn et al. 2014).

### Preparation of cell lysates

WT and *cobB* deletion mutants were grown in 15 mL LB at 37**°**C overnight with shaking at 200 rpm in the absence of antibiotic, or presence of 34 *μ*g/*μ*L chloramphenicol, respectively. These cultures were used to inoculate 500 mL of TB and were grown to an *A*_600_ of 0.8 at 37**°**C with shaking at 200 rpm. Cells were pelleted by centrifugation and resuspended in 50 mL buffer (25 mmol/L Tris pH 8.0, 137 mmol/L NaCl, 2.7 mmol/L KCl, 0.5 mmol/L DTT) with one complete protease inhibitor tablet (Roche Applied Science, Madison, WI, USA). Cells were sonicated, centrifuged, and the supernatant was removed for analysis.

### SAMDI reactions with cell lysates

To prevent interactions of DNA and RNA present in the lysate with the positively charged peptide surfaces of the SAMDI biochips, lysates were treated with benzonase (≥99% pure, 100,000 U/vial Millipore, Billerica, MA, USA). Five milliliters of lysate were incubated with 2 *μ*L of benzonase (∼500 U) and 10 mmol/L MgCl_2_ at 37**°**C for 30 min. Each lysate was filtered using an acrodisc 0.2 *μ*mol/L filter and was diluted 1:1 with assay buffer plus 0.5 mmol/L final concentration of NAD. Three microliters of extract were incubated on each spot on the SAMDI biochips containing Ac-GXK(Ac)ZGC-CONH_2_ peptides at 30**°**C for 2 h and reactions were terminated and analyzed as described above. Spectra were manually inspected to verify activity.

### Structural analysis of CobB substrate proteins

All structures of CobB substrate proteins were downloaded from the Protein Data Bank (PDB) and were manually inspected in pymol to determine residues adjacent to the substrate lysine in 3D space. When *E. coli* structures were not available, homologous structures containing the conserved substrate lysine residue were used (for description of structures used for analysis, see Table S6). The 1D and 3D sequences of the CobB substrate proteins surrounding the acetylated lysine were compared with the SAMDI profile of recombinant CobB (listed in Tables S7 and S8). Figures were produced using pymol (DeLano 2002).

### Western immunoblot analysis of cell lysates

Overnight cultures were diluted into fresh TB7 at an OD600 of 0.1. Cells were harvested at OD600s of 0.5 and 1.0 and then at hours 8, 24, and 32. Cells were pelleted and lysed with 4× sample buffer (9% SDS [sodiumdodecyl sulfate], 0.36 mol/L Tris pH 6.8, 45% glycerol, 2% bromophenol blue, 10% *β*-mercaptoethanol) and heated at 100°C for 10 min. Protein loading was normalized after protein amount was determined by BCA assay (23225; Pierce, Rockford, IL, USA). Purified proteins were separated by SDS-PAGE (polyacrylamide gel electrophoresis) with 4.6 mol/L urea. The gel and the nitrocellulose membrane were rinsed in water and then equilibrated for 15 min in transfer buffer containing 25 mmol/L Tris, 0.192 mol/L glycine, and 20% (v/v) methanol. Proteins were transferred onto the membrane for 1 hour at 100 V. The blot was blocked with 5% (w/v) milk prepared in TBST for 2 h at room temperature. The blot was washed with PBST two times for 5 min each. A rabbit polyclonal antibody raised against an acetylated lysine-containing peptide (9441; Cell Signaling, Danvers, MA, USA) was used at a 1:500 dilution in 5% BSA in PBST at 4°C overnight. The blot was washed three times for 5 min each and then one time for 10 min with PBST and then incubated with horseradish peroxidase (HRP)-conjugated goat anti-rabbit secondary antibody (7074S; Cell Signaling) at a 1:2000 dilution in 5% milk in TBST for 1 hour at room temperature. The blot was washed four times for 5 min each with PBST and exposed using 20× LumiGLO® Reagent and Peroxide (7003; Cell Signaling).

### Identification of lysines acetylated in the *ackA cobB* mutant

The *ackA cobB* mutant was grown aerobically at 37°C at 250 rpm in TB7 until entry into stationary phase, cell lysates were prepared and the proteins were separated by SDS-PAGE, as described (Hu et al. 2013). A band that was observed in the *cobB ackA* mutant but not in either single mutant or the WT parent was excised, subjected to tryptic digestion, peptides eluted and subjected to reversed phase column chromatography, as described (Hu et al. 2013). MS and MS/MS data were acquired with the LTQ-Orbitrap-Velos mass spectrometer (Thermo Fisher Scientific, Waltham, MA, USA) equipped with a nanoelectrospray ion source and the acetyllysines identified, as described (Hu et al. 2013).

## Results

### CobB-dependent deacetylation occurs in diverse cellular processes

From cells grown in buffered tryptone broth (TB7) supplemented with 0.4% glucose, our quantitative mass spectrometric analysis (Kuhn et al. 2014) identified 69 acetyllysine sites on 51 proteins, whose relative abundance was robustly, reproducibly, and significantly elevated in the *cobB* mutant relative to WT (defined as a ≥twofold acetylation increase in at least three of four biological replicates with a *P* < 0.05) (Table S1). To examine the potential biological impact of this CobB-dependent deacetylation, we performed functional analyses using PANTHER (Mi et al. 2013). The most common molecular function of proteins containing the preferred acetyllysine targets was “catalytic activity,” but the functions “binding” and “structural molecule” activity also were common (Fig. S1A, Table S2A). By far, the most common biological process was “metabolic process” (Fig. S1B, Table S2B). Enrichment analysis by DAVID (Huang da et al. 2007) (Table2) supports the hypothesis that CobB-dependent deacetylation primarily targets translation (“protein synthesis,” “ribosome,” “ribonucleoprotein,” “RNA-binding,” “rRNA-binding,” “elongation factor,” and “initiation factor”) and acetylated proteins (“acetylation”), but also some DNA-centered processes (“DNA binding,” “DNA condensation,” “activator,” “transcription,” and “transcription regulation”), phosphorylated proteins “phosphoprotein,” and central metabolism (“glycolysis” and “pyridoxal phosphate”). Kegg pathway (Table S2C) and Gene Ontology (Table S2D) analyses yielded similar results, with a focus on translation, central metabolism, and DNA-centered processes. Others have observed similar results, although a direct comparison cannot be made due to differences in growth conditions (Weinert et al. 2013).

**Table 2:** Functional annotation enrichment for 51 acetylated proteins with *cobB*-sensitive acetyllysine sites

Term^1^	Count^2^	%^3^	Fold enrichment^4^	*P*-value^4^	Bonferroni
Protein biosynthesis	15	25.9	77.2	8.4E-23	9.9E-21
Ribosome	10	17.2	146.2	1.9E-17	2.3E-15
Ribonucleoprotein	10	17.2	107.7	3.5E-16	3.9E-14
Ribosomal protein	10	17.2	102.3	5.7E-16	6.6E-14
Acetylation	12	20.7	46.6	1.6E-15	1.8E-13
RNA-binding	7	12.1	30.8	1.0E-07	1.2E-05
DNA binding	7	12.1	27.7	1.9E-07	2.3E-05
Phosphoprotein	8	13.8	18.5	2.4E-07	2.8E-05
Cytoplasm	13	22.4	6.0	9.9E-07	1.2E-04
DNA condensation	4	6.9	192.6	1.1E-06	1.3E-04
rRNA-binding	4	6.9	64.2	3.3E-05	0.004
Activator	5	8.6	17.3	1.9E-04	0.022
DNA binding	11	19.0	4.1	2.6E-04	0.030
Elongation factor	3	5.2	74.4	7.3E-04	0.083
Glycolysis	3	5.2	43.9	0.002	0.219
Methylated amino end	2	3.4	327.5	0.006	0.508
Transcription	8	13.8	3.5	0.007	0.553
Initiation factor	2	3.4	272.9	0.007	0.573
Duplication	2	3.4	272.9	0.007	0.573
Methylation	3	5.2	23.0	0.007	0.584
Methylated amino acid	2	3.4	204.7	0.010	0.678
Capsule biogenesis/degradation	2	3.4	181.9	0.011	0.721
Molecular chaperone	2	3.4	136.5	0.014	0.817
Heterodimer	2	3.4	102.3	0.019	0.896
Transcription regulation	7	12.1	3.1	0.024	0.941
Pyridoxal phosphate	3	5.2	10.5	0.032	0.980
Metal binding	6	10.3	3.2	0.039	0.991
Chaperone	3	5.2	9.3	0.040	0.992
Oxidoreductase	6	10.3	3.1	0.042	0.994

1 SP_Pir keyword/GO ontology term.

2 Number of DAVID ID's matching the specific GOTERM.

3 % of count matching a specific GO category over total number of DAVID ID's entered for analysis (58).

4 Because our peptide-based affinity enrichment strategy achieved a dynamic range of about 7 orders of magnitude, identifying acetylated lysines from proteins with very low, as well as very high, estimated protein copy numbers per cell (Kuhn et al. 2014), we used the the *E. coli* proteome as background.

### CobB-sensitive sites in the ribosome

**Figure 2:**
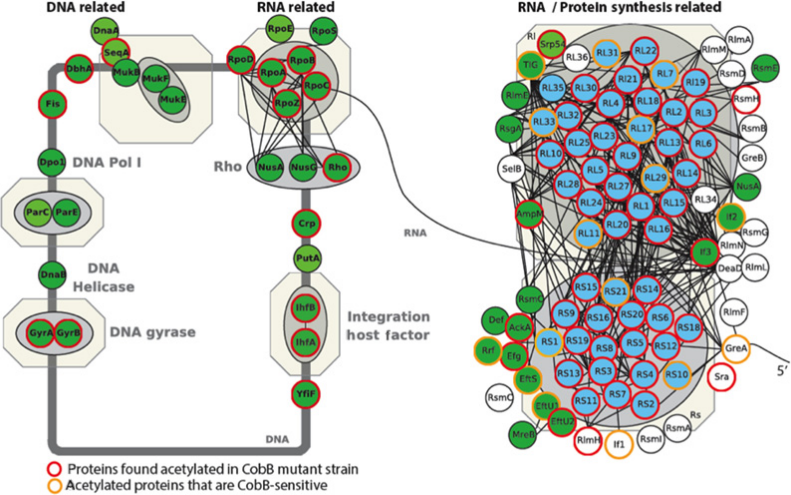
Schematic of *E. coli* ribosomal subunits and other protein synthesis-related proteins showing proteins with acetyllysines that are sensitive to CobB. Proteins identified as acetytated in *cobB* mutants are circled in red, proteins containing verified CobB-sensitive acetyllysines are circled in orange. In addition to ribosomal and protein synthesis-related proteins, other DNA and RNA pathways are also shown (see WikiPathways and (Papanastasiou et al. 2013). Note that Papanastasiou and coauthors did not detect ribosomal subunit S17 and thus it is not depicted in the figure.

Because CobB-dependent deacetylation was enriched for translation, we examined the CobB-sensitive lysines of ribosomal subunits. A total of 14 CobB-sensitive acetylation sites were identified on 10 ribosomal protein subunits as shown in Figure2, which maps onto a WikiPathway display (Papanastasiou et al. 2013) RNA/protein synthesis and other DNA- and RNA-related proteins with acetyllysines in the *cobB* mutant that were determined to be sensitive to CobB (yellow circles) or not (red circles) (for details, see Table S3A). The CobB-sensitive ribosomal proteins included 6 subunits of the 50S subcomplex (RplK [L11], RplL [L7/L12], RplQ [L17], RpmC [L29], RpmE [L31], RpmG [L33]) and 4 subunits of the 30S subcomplex (RpsA [S1], RpsJ [S10], RpsQ [S17], and RpsU [S21]) (Herold and Nierhaus 1987; Culver 2003; Shajani et al. 2011).

To determine if any ribosomal protein CobB substrate acetyllysines (Table S3B and C) were located on regions important for translation, we examined their structures within the full 70S *E. coli* complex. The structures of all ribosomal subunits were analyzed using either PDB ID: 3R8S or PDB ID: 4DG1. The exceptions were L31, which we analyzed using the PDB ID: 2VHM structure, and S1 and L7/L12, which were not found in these 70S structures. Only portions of the S1 structure have been determined and none of the structures include the CobB substrate lysines. The L7/L12 subunit is described in more detail below.

In our protein structure analysis, we determined that CobB-sensitive lysines of many of the ribosomal subunits (L11, L17, L29, L31, and S10) are located near 23S rRNA; however, these lysines make no obvious polar contacts. The substrate lysine of L33 (Lys-81) resides near the “E” site tRNA and is close to the 30S/50S subunit interface, but this lysine does not make polar contacts with RNA or with other subunits. In contrast, the CobB-sensitive lysine residues of the S17 and S21 subunits (Lys-71 and Lys-25, respectively) make direct polar contacts with RNA. Lys-71 of the S17 subunit forms hydrogen bonds with the backbone amide of Lys-19 and with the 16S rRNA backbone phosphate located between guanosines 254 and 255. The *ε*-amino group of Lys-25 of the S21 subunit forms a hydrogen bond with the 2′-OH of the ribose of guanosine 9 of mRNA in the crystal structure (mRNA chain X, 4GD1). The acetylation status of these CobB substrates could be expected to influence translation.

### Investigation of CobB substrate specificity

**Figure 3:**
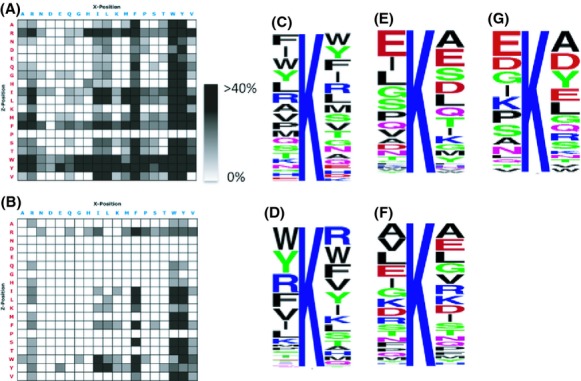
SAMDI MS peptide specificity profiles and LOGO plots for recombinant CobB protein and WT lysates. Activity of recombinant *E. coli* CobB (A) and of lysate from wild-type *E. coli* (strain MG1655; B) was determined using a GXKacZGC peptide library and SAMDI mass spectrometry. All data are the average of three separate trials (Table S4). Shading is indicative of percent conversion to product as shown. We did not detect any deacetylase activity in the lysate of the *cobB* deletion mutant (strain AJW5037). WebLogo (Crooks et al. 2004) was used to generate consensus sequence logos showing the amino acid composition in positions −1 to +1 relative to the recombinant CobB SAMDI peptide substrate lysines (C), the WT lysate SAMDI peptide substrate lysines (D), 69 *cobB*-sensitive lysines on 51 proteins (E), the 736 lysines on the same 51 proteins that were not *cobB*-sensitive (F), and the substrate lysines in 3D space (not including sequences where the adjacent residue could not be identified; see Table S7). SAMDI, self-assembled monolayers with matrix-assisted laser desorption-ionization.

To determine CobB substrate specificity, we measured the activity of purified recombinant CobB toward an acetylated peptide library using SAMDI mass spectrometry. This method, which monitors peptide modification of a peptide library immobilized onto a gold surface (Gurard-Levin and Mrksich 2008; Mrksich 2008; Gurard-Levin et al. 2009; Kuhn et al. 2014), found that recombinant CobB was very efficient and highly active with broad peptide substrate specificity (Fig.3A, Table S4). However, it preferentially deacetylated acetyllysines adjacent to hydrophobic and nonpolar residues, especially phenylalanine (F) and tryptophan (W), but also acetyllysines adjacent to positively charged arginine (R) and hydrophilic and polar tyrosine (Y) residues. In contrast, CobB did not deacetylate acetyllysines with a proline (P) in the Z- (i.e., +1) position, and had lower preferrence for acetyllysines with adjacent acidic residues, aspartate (D) or glutamate (E) residues (Fig.3C).

We next determined the substrate specificity of the deacetylase(s) present in a wild-type cell lysate. Here, we observed a more narrow substrate specificity (Fig.3B). However, the profile was similar to that of recombinant CobB, with a strong preference for tryptophan, tyrosine, arginine, and phenylalanine (Fig.3D). Once again, proline in the Z-position and acidic residues were disfavored. Importantly, when we tested the *cobB* mutant lysate, we observed no major deacetylase activity (Table S4), indicating that CobB is the predominant deacetylase, at least under these reaction conditions.

To see if the in vitro SAMDI-MS peptide deacetylation profile was recapitulated in vivo, we examined the protein primary sequences of the CobB-sensitive sites that our quantitative mass spectrometric analysis had identified (Kuhn et al. 2014). We first compiled a table of neighboring amino acids in the positions adjacent to each of the 69 acetyllysine sites in 51 proteins that were robustly, reproducibly, and significantly elevated in the *cobB* mutant. We then used WebLogo (Crooks et al. 2004) to generate a sequence logo (Fig.3E, Table S5). The deacetylation profile of CobB-sensitive acetyllysines identified by quantitative mass spectrometry was almost the opposite of the profile obtained from the in vitro SAMDI analysis with a high prevalence of negatively charged glutamate residues in the −1 (X) position (∼19%) and glutamate and aspartate in the +1 (Z) position (∼13% and 9%, respectively), and a low prevalence for arginine, phenylalanine, tryptophan, and tyrosine. Like SAMDI, this analysis showed a preference for no proline in the Z-position. A similar analysis, performed on 760 lysine residues from the same 51 proteins that were not detected as CobB-sensitive (Fig.3F, Table S5), revealed a somewhat different motif. Most notable was the reduced prevalence of adjacent negatively charged residues.

Since the in vitro and in vivo linear (1D) sequences did not match, we examined the native three-dimensional (3D) environment of CobB-sensitive acetyllysines. The 3D environment gives a more complete depiction of the arrangement of adjacent residues to the substrate lysines than SAMDI or in vivo linear sequence analyses, thus we used this information for subsequent analyses. For each protein with a CobB-sensitive acetyllysine, we analyzed a representative structure to determine the location of the substrate residue and its corresponding type of secondary structure. Of the 51 proteins that quantitative mass spectrometry identified as possessing CobB-sensitive acetyllysines, 43 had 3D structures in the Protein Data Bank (PDB). Of these 43 structures, 34 were from *E. coli* proteins, while the remaining nine were from bacterial homologs. These 43 proteins included 57 of the 69 CobB-sensitive acetyllysines (Table S6).

We analyzed these 43 individual crystal structures and identified the secondary structure in which each substrate acetyllysine resides (Table S6), as well as the amino acids adjacent to the substrate acetyllysine in 3D space (Table S7). Ten percent (6/57) of the substrate acetyllysines were located on *β*-strands, while 44% (25/57) were on *α*-helices, and 46% (26/57) were on loops. Thus, no strictly conserved secondary structure was identified across all CobB acetyllysine substrates. For some CobB substrate acetyllysines, the identities of the adjacent residues were unclear because they reside on disordered loops (9/57) or at the ends of alpha helices (9/57); thus, these substrate lysines were removed from the subsequent analysis, which was performed on the remaining (39/57) sites. For these 39 CobB substrate acetyllysines, we first used the crystal structures to identify the adjacent amino acid residues in 3D. Typically, the most abundant adjacent residues to the substrate lysine were aspartate (D), glutamate (E), alanine (A), glycine (G), and tyrosine (Y) residues (Fig.3G, Table S7), a pattern that showed some resemblance to the 1D linear in vivo sequence, especially with respect to the abundance of negatively charged residues (Fig.3E). We then compared the residues adjacent to the substrate lysines in the 3D structures to the 1D SAMDI profile obtained with recombinant CobB. Fifty-four percent (20/37) of the sequences matched the SAMDI profile (Table S8) with substrate lysines located on *β*-sheets having the highest correlation with the SAMDI profile (67%, 4/6), followed by loops (65%, 11/17) and *α*-helices (36%, 5/14); two sequences could not be compared because their identity in 3D was unclear). We conclude that secondary structure influences the ability of CobB to recognize its substrate acetyllysines and thus reliance on primary sequence alone to identify such substrates is insufficient.

**Figure 4:**
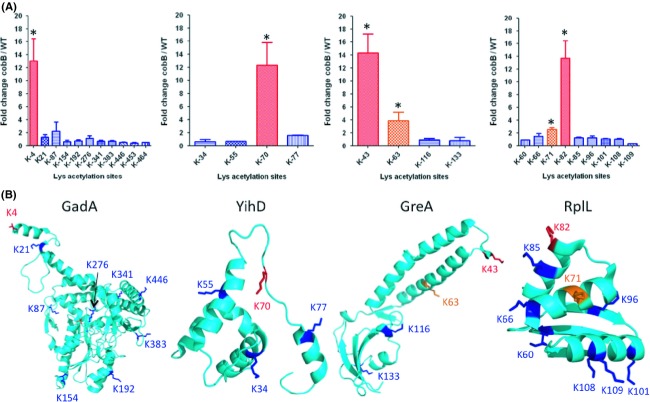
Correlation of CobB-sensitive acetyllysines to protein structure. (A) Mass spectrometric quantification of relative abundance of acetyllysine-containing peptides and their fold change between the *cobB* mutant (strain AJW5037) and its WT parent (strain MG1655) grown in TB7 supplemented with 0.4% glucose. Data were obtained from four independent biological replicates and three technical replicates. Bar graphs show the average fold change (*cobB*/WT) for individual acetyllysine sites in the proteins GadA, YihD, GreA, and RplL. Acetyllysine sites with the most significant CobB-sensitive upregulation are highlighted in red, sites with significant upregulation in orange, and sites with relatively no change in blue. (B) *E. coli* crystal structures of these proteins (from PDB) are visualized using Pymol, indicating the lysine residues whose acetylation was monitored by mass spectrometry and shown in (A). Crystal structures for each protein are shown in cyan (PDB ID: chain A of 1XEY (GadA), 2KO6 (YihD), 1GRJ (GreA), and 1CTF (RplL)). Acetyllysine sites with the most significant CobB-sensitive upregulation are highlighted in red, sites with significant upregulation in orange, and sites with relatively no change in blue. In GadA, a portion of the K4 residue is disordered and residues K453 and K464 are not present in structure. PDB, Protein Data Bank.

Therefore, to further analyze the relationship between the position of a lysine within its protein structure and its sensitivity to CobB, we selected four proteins that contained at least one specific lysine that was robustly, reproducibly, and significantly more acetylated in the *cobB* mutant relative to WT: GadA, GreA, RplL, and YihD (Fig.4). In each case, structural analysis showed that the CobB-sensitive site(s) were exposed to the surface. A particularly dramatic example is K4 of GadA, which is located on the tip of a protruding helix. Similarly, K43 of GreA is located in an exposed hairpin between two helices. Another GreA lysine (K63) resides halfway down the side of one of those helices. Other GreA lysines that were acetylated, but did not show sensitivity to CobB (K116 and K133), were located in positions that appeared to be less exposed. In these cases, CobB substrate acetyllyines appear to be located near the surface of the protein on a loop or a helix that protrudes into the solution and thus in position to be an easily accessible target.

### Evidence that CobB can deacetylate lysines acetylated by acP

**Figure 5:**
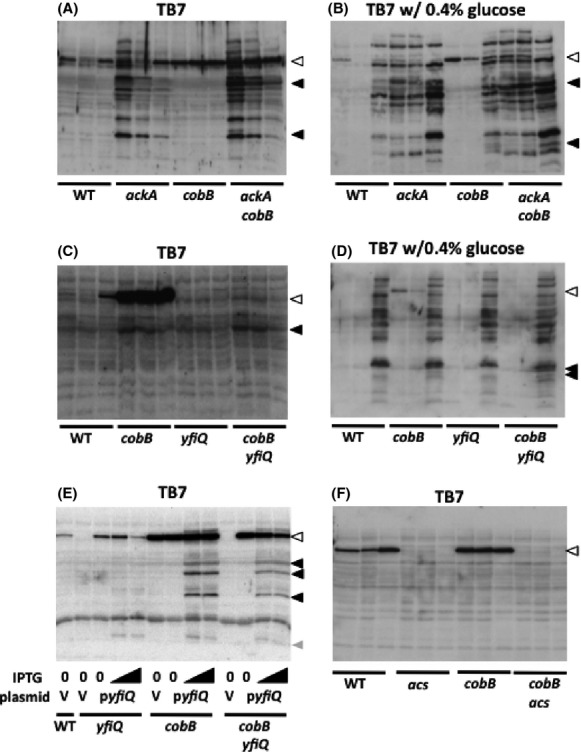
Antiacetyllysine Western immunoblot analyses. Cells were aerated at 37°C in TB7 (A, C, F), in TB7 supplemented with 0.4% glucose (B, D), or in TB7 supplemented with 25 *μ*g/mL chloramphenicol (E) and harvested at three time points, when the OD_610_ reached 0.5 or 1.0, and then at 8 h. (A and B) *cobB ackA* epistasis analysis: *E. coli* WT (strain AJW678) and isogenic mutants *ackA* (strain AJW2067), *cobB* (strain AJW4343), and *ackA cobB* (strain AJW5024). The white arrow points to an ∼70-kDa band that is sensitive to CobB and less prominent in glucose. The black arrows point to bands that are observed only in the *ackA cobB* double mutant. (C and D) *cobB yfiQ* epistasis analysis: WT (strain AJW678) and isogenic mutants *cobB* (strain AJW4343), *yfiQ* (strain AJW4344), and *cobB yfiQ* (strain AJW5121). The white arrow points to an ∼70-kDa band that is sensitive to YfiQ and CobB. The black arrows point to bands that are observed only in the *cobB yfiQ* double mutant. (E) YfiQ overexpression: WT (strain AJW678) transformed with the vector pCA24n (strain AJW5126), the *yfiQ* mutant (strain AJW4344) transformed with the vector pCA24n (strain AJW5130) or with the YfiQ expression plasmid pCA24n-*yfiQ* (strain AJW5131), the *cobB* mutant (strain AJW4343) transformed with the vector pCA24n (strain AJW5129) or with the YfiQ expression plasmid pCA24n-*yfiQ* (strain AJW5124), and the double *cobB yfiQ* mutant (strain AJW5121) transformed with the vector pCA24n (strain AJW5128) or with the YfiQ expression plasmid pCA24n-*yfiQ* (strain AJW5125). YfiQ expression was induced by IPTG (15 or 25 *μ*mol/L). The white arrow points to an ∼70-kDa band that is sensitive to YfiQ and CobB. The black arrows point to 3 more YfiQ-sensitive bands that depend on CobB for deacetylation. The gray arrow points to one YfiQ-sensitive protein that is not sensitive to CobB. (F) *acs cobB* epistasis analysis: *E. coli* WT (strain AJW678), and isogenic mutants *acs* (strain AJW1781), *cobB* (strain AJW4343), and *acs cobB* (strain AJW5185). The white arrow points to an ∼70-kDa band that is sensitive to CobB and depends on Acs.

To explore the relationship between CobB-dependent deacetylation and acP-dependent acetylation, we performed an antiacetyllysine Western immunoblot analysis on wild-type (WT) cells and isogenic mutants that lack CobB (*cobB*), accumulate acP (*ackA*), or both (*ackA cobB*). As we reported previously (Kuhn et al. 2014), the *ackA* mutant exhibited increased acetylation of large numbers of proteins when grown in TB7 (Fig.5A). In contrast, the *cobB* mutant exhibited no obvious change in the level of protein acetylation, although it did show an increase in acetylation of a particularly prominent band (open arrowhead). The *ackA cobB* double mutant exhibited both patterns, consistent with the idea that acP and CobB regulate acetylation of different sets of proteins. Intriguingly, the double mutant also exhibited two bands that were not visible in both single mutants (solid arrowheads). This is consistent with the hypothesis that some proteins acetylated by acP can be deacetylated by CobB. A similar relationship was observed when the same strains were grown in TB7 supplemented with 0.4% glucose (Fig.5B). For the indicated gel band (upper black arrowhead) of the *ackA cobB* double mutant, MS analysis identified many proteins, of which four were acetylated (EF-Tu, Pyk, Fba, and GapA) on a total of 9 lysines (Table S9A–D). When we correlated these results with the corresponding quantitative mass spectrometry data set described above (Kuhn et al. 2014), we found that five of these acetyllysines were significantly upregulated in the *ackA* mutant relative to the WT parent. These data suggest that CobB can deacetylate lysines that are primarily acetylated by acP.

To obtain biochemical evidence that CobB can indeed reverse acP-dependent acetylation, we used a SAMDI biochip containing a library of unacetylated lysine-containing peptides. We incubated the biochip first with acP and then with recombinant CobB, which completely deacetylated all peptides that had been modified by acP (data not shown).

To further explore the relationship between acP-dependent acetylation and CobB-dependent deacetylation, we more thoroughly examined the quantitative mass spectrometric data that had been obtained from WT, *ackA*, and *cobB* cells grown in TB7 supplemented with glucose and harvested upon entry into stationary phase (equivalent to Fig.5B, lanes 3, 6, and 9). Because CobB-dependent deacetylation was enriched for translation, we first examined CobB-sensitive lysines in ribosomal subunits. As stated earlier, 14 lysines of 10 ribosomal subunits were sensitive to CobB. In contrast, 80 lysines of 40 subunits were sensitive to AckA (Fig.2, Table S3A and B). In support of the hypothesis that acP can acetylate and CobB can deacetylate the same proteins, all 10 of the CobB-sensitive subunits included lysines that also were sensitive to AckA. Strikingly, four of these subunits (RplL [L7/L12], RpmE [L31], RpmG [L33], and RpsU [S21]) had a total of six lysines that were sensitive to both CobB and AckA (Table S3C).

This general pattern was observed for the entire set of CobB-sensitive proteins and acetyllysines. Of the 51 proteins that possessed a CobB-sensitive acetyllysine, 40 had at least one lysine whose acetylation depended on the status of AckA. Together, these 40 proteins had 58 CobB-sensitive acetyllysines: 24 also were sensitive to AckA, while 34 were not detected as AckA-sensitive (Table1). Thus, some proteins were sensitive to CobB but not AckA, while other proteins were sensitive to both CobB and AckA, but on different lysines. Importantly, one subset of proteins possessed lysines that were sensitive to both CobB and AckA. Proteins in this last class included the 4 ribosomal subunits listed above, two chaperones (DnaK and GroS), and two central metabolic subunits (AccA and LpdA). This last class also included the two-component response regulator RcsB. More specifically, CobB and AckA regulated acetylation of K154 (Kuhn et al. 2014), which we had previously implicated in transcription of the small RNA RprA (Hu et al. 2013). These results are consistent with the hypothesis that some lysines are substrates for acP, while others are substrates for CobB, and still others are substrates for both.

### Evidence that CobB can deacetylate lysines acetylated by YfiQ

To verify that CobB can also deacetylate targets of YfiQ, the only known *E. coli* acCoA-dependent protein acetyltransferase, we first tried to identify the prominent CobB-sensitive (and AcP-insensitive) band (Fig.5, open arrowhead). WT cells and isogenic mutants that lack either YfiQ (*yfiQ*) or CobB (*cobB*) or both (*yfiQ cobB*) were grown in TB7 or TB7 supplemented with 0.4% glucose. Antiacetyllysine Western immunoblot analysis of cells grown in the absence of glucose again detected the prominent band, indicative of a protein of ∼70 kDA, whose acetylation and deacetylation appeared to depend on YfiQ and CobB, respectively (Fig.5C). This behavior did not depend on genetic background (Fig. S2A) or particular *yfiQ* null allele (Fig. S2B), nor did it depend on the presence of glucose, although the signal was less intense (Fig.5D). We verified the dependence on YfiQ by transforming cells with either a YfiQ expression plasmid or its vector control and growing the resultant transformants in TB7 supplemented with or without IPTG to induce YfiQ expression (Fig.5E). This YfiQ-dependent and CobB-sensitive signal depends on AcCoA-synthetase (Acs), as the signal was missing in cells that lacked both Acs and CobB (Fig.5F). Other less prominent acetylation targets appeared to be regulated by YfiQ and/or CobB: these immunoblots suggest that some acetylation events are independent of YfiQ but sensitive to CobB (Fig.5C and D), while several depend on YfiQ overexpression and the absence of CobB (Fig.5E).

## Discussion

We conclude that CobB is the predominant lysine deacetylase in *E coli*, that CobB can deacetylate acetyllysines independently of whether the acetyl donor is acCoA or acP, and that CobB deacetylates proteins involved in several key cellular processes. We further conclude that linear sequences alone are inadequate to predict CobB substrates substrates and that structural analyses are necessary.

### CobB is the predominant deacetylase in *E. coli*

An unanswered question has been whether *E. coli* expresses another deacetylase in addition to CobB. To date, there is no evidence that such a deacetylase exists. Indeed, our in vitro SAMDI experiment provided evidence that CobB is the predominant deacetylase: in contrast to the WT cell lysate (Fig.3), the *cobB* mutant lysate exhibited virtually no deacetylase activity above assay background (Table S4). *Salmonella enterica* expresses CobB in two isoforms, one short and one long (Tucker and Escalante-Semerena 2010). Based on bioinformatic inspection of its genome, the same should be true of *E. coli*. But, the expression of two isoforms cannot explain our results, as the WT lysate would be expected to contain both forms, while the *cobB* lysate should contain neither.

### CobB deacetylates some acP-dependent acetyllysines, but not others

The lack of evidence for an additional deacetylase raises an intriguing problem. The number of acetylated lysines vastly outnumbers the number of CobB acetyllysine substrates. For example, we recently showed that acP can robustly donate its acetyl group to 592 lysines on 292 proteins (Kuhn et al. 2014). In contrast, CobB targets only 69 acetyllysine sites on 51 proteins (Table S1) and only 24 lysines are sensitive to both acetylation by acP and deacetylation by CobB (Table1, Table S3C). Thus, CobB does not significantly reverse the vast majority of acP-dependent acetylations. A similar scenario has been reported in mouse liver mitochondria, where nonenzymatic acetylation by acCoA (presumably driven by the elevated pH and high acCoA levels in this organelle) has been proposed to be the dominant mechanism (Rardin et al. 2013; Wagner and Payne 2013) and the sirtuin deacetylase SIRT3 effectively targets a small fraction (∼20%) of the total known acetyllysine sites (Sol et al. 2012; Hebert et al. 2013; Rardin et al. 2013).

It is distinctly possible that the 24 lysines described above represents a subset of the total of acP- and CobB-sensitive lysines. The *ackA cobB* double mutant, which accumulates acP and thus acP-dependent acetylation and lacks the ability to deacetylate via CobB exhibited additional signals in an antiacetyllysine Western blot (Fig.5A and B). This provides evidence that CobB can deacetylate some acetyllysines that occur when intracellular acP accumulates, which occurs during growth on excess glucose and other carbon sources that enhance fermentation. Since we did not observe these signals in cells with intact CobB (Fig.5A and B), we infer that CobB can reverse acetylation by acP. If so, then CobB would perform two distinct functions: it would reverse specific regulatory acetylations (e.g., Acs acetylation) (Starai et al. 2002), and it would “clean up” after acP, removing acetyl groups from some acetyllysines but not others.

### CobB deacetylates acetyllysines regardless of the acetyl donor

In addition to a subset of acP-dependent acetyllysines, CobB can deacetylate some, but not all, YfiQ- and acCoA-dependent acetyllysines. The only known YfiQ substrate that is also well established as a CobB substrate is Acs, whose acCoA-synthesizing activity is inhibited by acetylation of K609 (Starai and Escalante-Semerena 2004) and reactivated by its CobB-catalyzed deacetylation (Starai et al. 2002). Others have reported a few additional proteins (GapA, AceA, and AceK) that may be bonafide YfiQ and CobB substrates (Wang et al. 2010), but the reversible acetylated lysines have not been identified. The chemotaxis protein CheY has been reported to be a CobB substrate (Li et al. 2010), but there is no evidence that YfiQ acetylates it and the CobB substrate acetyllysine has not been identified. In contrast, Lys-544 of RNase R is acetylated by YfiQ (Liang et al. 2011), but not deacetylated by CobB (Liang and Deutscher 2012). The rapid degradation of the acetylated form of RNase R might preclude the need for deacetylation. While other acetylated, but not deacetylated, proteins might be similarly sensitive to degradation, this is likely not true of the vast majority of acetylated proteins that are not sensitive to CobB. This raises an intriguing question: how do cells cope with acetylations that are not reversed by CobB?

Our results support the established hypothesis that YfiQ and CobB regulate the acetylation status of Acs and provide evidence for the existence of other dual regulated substrates. Our immunoblot analysis revealed an ∼70 kDa signal that increased in the absence of CobB, depended strongly upon YfiQ and Acs, and diminished during growth in glucose (Fig.5). These are all behaviors expected of a catabolite repressible gene (Kumari et al. 2000), whose 72 kDa product is acetylated by YfiQ (Starai and Escalante-Semerena 2004) and deacetylated by CobB (Starai et al. 2002). Since several signals appeared when YfiQ was overexpressed in the absence of CobB (Fig.5E), it is also possible that CobB deacetylates inappropriate YfiQ-dependent acetyllysines, as proposed above for acP. The identities of these putative substrates remain to be determined.

### CobB's effect on protein function

Enrichment analyses (Table2, Table S2) support the hypothesis that CobB-dependent deacetylation primarily targets translation. Whether CobB deacetylation (or acP-dependent acetylation) of ribosomal subunits plays a role in ribosomal function and thus cellular physiology remains unknown; however, some clues exist. First, CobB-sensitive acetyllysines on two ribosomal subunits interact with RNA. Lys-71 of the S17 subunit interacts with 16S rRNA, while Lys-25 of the S21 subunit can interact with the mRNA. Second, two CobB-sensitive acetylated lysines on the L7/L12 (RplL) subunit could influence aminoacylated tRNA binding to the A-site and thus the rate of peptide elongation. Lys-71 and Lys-82, respectively, reside in helices 4 and 5 (Fig.4), which interact with helix D of elongation factor Tu (EF-Tu). Mutation of strictly conserved residues along these helices decreased the initial binding association rate constant, but not the dissociation rate constant (Kothe et al. 2004). Thus, acetylation of Lys-71 could affect L7/L12 binding to EF-Tu, an event that facilitates EF-Tu-dependent binding of charged tRNA to the A-site. Since Lys-82 of helix 5 is not strictly conserved, Kothe and coworkers did not mutagenesize this residue. However, they mutagenized another lysine (Lys-85), which resides on the same face of helix 5 and forms a salt bridge with D141 of helix D of EF-Tu. The effect was nearly identical to that of the Lys-71 mutation. Thus, the acetylation status of Lys-71 and/or Lys-82 could determine the association rate of the ternary complex, but may also play an important role in binding to other proteins. Further testing is necessary to determine the biological consequence of acetylating these residues.

Bioinformatics ontology and pathway enrichment analyses (Tables2 and S2) also support the hypothesis that CobB-dependent deacetylation targets central metabolism and phosphorylated proteins, and some DNA-centered processes. As mentioned earlier, others have reported that CobB deacetylates Acs (Starai et al. 2002) and a few other central metabolic enzymes (Wang et al. 2010), as well as CheY (Li et al. 2010) and RcsB (Thao et al. 2010), two-component response regulators that become activated when phosphorylated. We further reported (1) that transcription from the *rprA* promoter requires Lys-154 of RcsB (Hu et al. 2013), (2) that the acetylation status of Lys-154 is regulated by acP and CobB (Hu et al. 2013; Kuhn et al. 2014), and (3) genetic evidence consistent with the hypothesis that acetylation of Lys-154 inactivates RcsB (Hu et al. 2013).

GreA possesses two acetylated lysines (Lys-43 and Lys-63) that are sensitive to CobB. GreA is a transcript cleavage factor that prevents arrest during transcription elongation, increases transcription fidelity, stimulates promoter escape, and suppresses promoter proximal pausing during RNA synthesis (Fish and Kane 2002; Laptenko et al. 2003; Nickels and Hochschild 2004; Borukhov et al. 2005). GreA's N-terminal coiled-coil domain fits within a secondary channel of RNA polymerase (RNAP). This places two negatively charged residues (Asp-41 and Glu-44), located at the tip of the coiled-coil domain, in close proximity to an essential Mg^2+^ ion at RNAP's catalytic center. The size and orientation of the coiled-coil domain residues, in particular the placement of the residues at the tip, are critical for catalysis and interaction with RNAP (Laptenko et al. 2003). Mutation of these and other tip residues decreases GreA activity, without affecting RNAP binding (Laptenko et al. 2003). Removal of the functional group of Lys-43 beyond the *β* carbon (by mutation to alanine) did not significantly affect transcript elongation in vitro (Laptenko et al. 2003), suggesting that Lys-43 either plays no significant role in GreA function or that its influence depends on maintenance of the functional group, such that its charge matters. It is easy to imagine that a positive charge (Lys-43) located adjacent to a negative charge (Glu-44) that coordinates an essential catalytic Mg^2+^ ion could influence catalytic function and that neutralization of that charge by acetylation could be used to regulate function. Lys-43 is not conserved in the GreA homologs GreB and DksA, whose coiled-coil domains also fit within the secondary channel (Laptenko et al. 2003). These proteins perform similar, but not identical, functions that are determined by the identity of the tip residues (Rutherford et al. 2007). Further testing is necessary to determine the biological consequence of acetylating Lys-43.

GadA, a glutamic acid decarboxylase that helps bacteria survive extreme acidic stress (De Biase et al. 1999), contains a CobB-sensitive acetylated lysine (Lys-4). GadA exists as a dimer, but may also adopt a hexameric form. The helix on which Lys-4 resides helps to stabilize the hexamer (Dutyshev et al. 2005). To our knowledge, the role of Lys-4 in GadA has not been described and thus the biological effect of acetylating this residue remains unknown.

### CobB substrate specificity

CobB selectively deacetylates certain acetyllysine sites but not others, even within the same protein (Tables1 and S3) (Kuhn et al. 2014). We reported similar effects for mammalian SirT3 (Rardin et al. 2013). What determines specificity? One characteristic is surface accessibility. CobB-sensitive acetyllysines tended to be located near protein surfaces, most often on a loop or an *α*-helix that protruded into solution (Fig.4, Tables S6 and S8). Such residues should be easily accessible to an enzyme. This requirement for accessibility is one obvious reason why some less surface-exposed acetyllysines, dependent only upon the small molecule acP for their acetylation, might not be CobB targets.

Another characteristic is the local chemical environment. High-throughput peptide array (SAMDI) experiments showed that CobB, in either the purified recombinant form or the native form within cell lysate, favored acetyllysine substrates adjacent to residues that are large hydrophobic and nonpolar (Phe and Trp), positively charged (Arg), or large hydrophilic and polar (Tyr), while disfavoring substrates adjacent to prolines and residues that are acidic and negatively charged (Asp and Glu) (Fig.3A–D). Inspection of CobB-sensitive acetyllysines, as determined by quantitative mass spectrometry, revealed an almost opposite profile (Fig.3E). Examination of the 3D structure of CobB acetyllysine substrates found a pattern similar, but not identical, to that obtained by quantitative mass spectrometry: most often the residues adjacent to the substrate lysine were aspartate, glutamate, alanine, glycine, and tyrosine residues (Fig.3G, Table S7). Because examination of 1D and 3D motifs yielded different results, we consider the use of primary sequence alone inadequate to make predictions concerning CobB substrate specificity. Instead, we recommend the use of crystal structures to analyze the 3D environment of the CobB substrate lysine.

CobB showed rather broad substrate specificity in the SAMDI assay (Fig.3A). This broad specificity could be due to the fact that SAMDI uses small peptides that could interact favorably with the hydrophobic region near CobB's active site. Furthermore, small peptides have fewer constraints compared to full-length proteins, which contain more residues and surface area. Full-length proteins also can undergo conformational changes that could control substrate recognition. Indeed, peptides and proteins have distinct binding modes to CobB (Zhao et al. 2004). It is, therefore, likely that distal interactions between CobB and its protein substrates also control specificity in addition to residues directly adjacent to the acetylated lysine (Zhao et al. 2004).

## Concluding Remarks

CobB appears to be a flexible enzyme. CobB can deacetylate acetyllysines without regard for the acetyl group donor (this study) and it can reverse *N*^*ε*^-lysine succinylation (Colak et al. 2013). CobB appears to be the predominant, or perhaps only, KDAC in *E. coli*. CobB appears to deacetylate only a fraction of the acetyllysines detected by quantitative MS. Taken together, these observations beg the question: how does the cell cope with large numbers of acetylations that cannot be reversed? Finally, our findings may have broad impact, as many species are predicted to express CobB homologs (Hildmann et al. 2007).

## Supporting Information

**Figure S1.** Ontology assessment of *cobB*-sensitive acetyllysine sites. Fifty-one proteins that exhibited significant upregulation of 69 acetyllysine sites in the *cobB* mutant relative to WT were subjected to ontology analysis using Panther. (A) Molecular function GO categories, and (B) Biological processes GO categories. **Figure S2.** Anti-acetyllysine Western immunoblot analyses. (A) *E. coli* WT (strain MG1655) and isogenic mutants *cobB* (strain AJW5037), *yfiQ* (strain AJW5184), and *cobB yfiQ* (strain AJW5118) were aerated at 37°C in TB7 and harvested at 3 time points, when the OD_610_ reached 0.5 or 1.0, and then at 8 h. The white arrow points to a 70-kDa band that depends on YfiQ and CobB. (B) WT (strain AJW678), the *cobB* mutant (strain AJW4343), the *yfiQ* mutant (strain AJW4344), and the double *cobB yfiQ* mutant (strain AJW5109) were aerated in TB7 at 37°C and harvested at three time points, when the OD_610_ reached 0.5 or 1.0, and then at 8 h. The white arrow points to a 70-kDa band that depends on YfiQ and CobB.

**Table S1.** Acetyl sites that are significantly regulated in the *cobB* mutant relative to WT. Defined as ratio >2 with *P* < 0.05 in at least three out of four biological replicates.

**Table S2.** Ontology analysis of proteins with CobB-regulated acetyllysines. (A) Panther ontology analysis, (B) pathway enrichment analysis, KEGG pathways, and (C) pathway enrichment analysis, gene ontology.

**Table S3.** Analysis of acetylated ribosomal proteins. (A) Ribosomal proteins with acetylated peptides that were significantly upregulated in the *cobB* mutant relative to the WT mutant. (B) Ribosomal proteins with acetylated peptides that were significantly upregulated in the *cobB* mutant relative to the WT parent (top) or significantly upregulated in the *ackA* mutant relative to the WT parent (bottom). (C) Acetyllysines that were upregulated in either the *cobB* mutant, the *ackA* mutant or both.

**Table S4.** The average deacetylase activity of recombinant CobB and lysates (WT and *cobB* deletion mutant) toward an acetylated peptide library using the SAMDI technique.

**Table S5.** Analyzing the residues neighboring CobB-sensitive and not sensitive lysines. Sixty-nine sites that exhibited significantly upregulated acetylation (ratio >2 with a *P* < 0.05 in at least three out of four biological replicates) in the *cobB* mutant relative to WT were analyzed. The amino acid frequencies were determined for the 51 proteins that had significantly upregulated acetyllysine modifications. The frequencies of the residues in the −1 and +1 positions relative to both the 69 CobB-sensitive and 760 non-CobB-sensitive lysines were determined. The frequencies of each amino acid neighboring these lysines were normalized to the frequencies of each amino acid in the 51 proteins. Ten lysine residues that were the terminal amino acid of a protein were not included in this analysis due to these residues lacking a + 1 position.

**Table S6.** Protein structures from the Protein Data Bank (PDB) used for 3D analysis of CobB substrate proteins. Substrate lysines for each protein identified by quantitative mass spectrometry are shown, as well as their corresponding lysine numbering in the structure. Homologous structures were used if no structure for *E. coli* had been determined. The type of secondary structure that contains the substrate lysine is also indicated.

**Table S7.** Amino acid residues adjacent to CobB substrate lysines in 3D. For candidate CobB substrates, amino acid residues that are adjacent to the substrate acetyllysine in 3D are shown. When appropriate, the residues from homologous structures are shown, but the corresponding residues in *E. coli* were used for further analysis. Yellow highlight indicates the substrate lysine residue, while cyan highlights the adjacent residues identified from 3D structures. LuxS from *Bacillus* has an insertion prior to the substrate acetyllysine that is not present in *E. coli*. Therefore, the corresponding adjacent residue in this region of the protein is not clear. If the identity of the residue adjacent to the acetyllysine was not clear from the structure, that residue is indicated with “X.”

**Table S8.** Correlation of adjacent residues to the CobB substrate acetyllysine in 1D versus in 3D. To determine the correlation, the three amino acids in 3D were compared with the SAMDI deacetylation profile for recombinant CobB (Fig. 3). An “X” indicates that the residue adjacent to the acetyllysine was unclear. A question mark indicates those 3D sequences whose correlations could not be determined due to the absence of an adjacent residue.

**Table S9.** Proteins with acetylated lysines found in the *cobB ackA* mutant and identified by mass spectrometry. A gel slice corresponding to the band indicated by the upper black arrow in Figure 5B was cut from a parallel SDS-polyacrylamide gel and subjected to mass spectrometry: (A) EF-Tu (TufA), (B) Pgk, (C) FbaA, and (D) GapA.

## References

[b1] Baba T, Ara T, Hasegawa M, Takai Y, Okumura Y, Baba M (2006). Construction of *Escherichia coli* K-12 in-frame, single-gene knockout mutants: the Keio collection. Mol. Syst. Biol.

[b2] Blander G, Guarente L (2004). The SIR2 family of protein deacetylases. Annu. Rev. Biochem.

[b3] Borukhov S, Lee J, Laptenko O (2005). Bacterial transcription elongation factors: new insights into molecular mechanism of action. Mol. Microbiol.

[b4] Browning DF, Beatty CM, Sanstad EA, Gunn KE, Busby SJW, Wolfe AJ (2004). Modulation of CRP-dependent transcription at the *Escherichia coli acs*P2 promoter by nucleoprotein complexes: anti-activation by the nucleoid proteins FIS and IHF. Mol. Microbiol.

[b5] Choudhary C, Kumar C, Gnad F, Nielsen ML, Rehman M, Walther TC (2009). Lysine acetylation targets protein complexes and co-regulates major cellular functions. Science.

[b6] Choudhary C, Weinert BT, Nishida Y, Verdin E, Mann M (2014). The growing landscape of lysine acetylation links metabolism and cell signalling. Nat. Rev. Mol. Cell Biol.

[b7] Colak G, Xie Z, Zhu AY, Dai L, Lu Z, Zhang Y (2013). Identification of lysine succinylation substrates and the succinylation regulatory enzyme CobB in *E. coli*. Mol. Cell Proteomics.

[b8] Crooks GE, Hon G, Chandonia JM, Brenner SE (2004). WebLogo: a sequence logo generator. Genome Res.

[b9] Culver GM (2003). Assembly of the 30S ribosomal subunit. Biopolymers.

[b10] De Biase D, Tramonti A, Bossa F, Visca P (1999). The response to stationary-phase stress conditions in *Escherichia coli*: role and regulation of the glutamic acid decarboxylase system. Mol. Microbiol.

[b11] DeLano WL (2002). Unraveling hot spots in binding interfaces: progress and challenges. Curr. Opin. Struct. Biol.

[b12] Dutyshev DI, Darii EL, Fomenkova NP, Pechik IV, Polyakov KM, Nikonov SV (2005). Structure of *Escherichia coli* glutamate decarboxylase (GADalpha) in complex with glutarate at 2.05 angstroms resolution. Acta Crystallogr. D Biol. Crystallogr.

[b13] Fish RN, Kane CM (2002). Promoting elongation with transcript cleavage stimulatory factors. Biochim. Biophys. Acta.

[b14] Gardner JG, Escalante-Semerena JC (2009). In *Bacillus subtilis*, the sirtuin protein deacetylase, encoded by the *srtN* gene (Formerly *yhdZ*), and functions encoded by the *acuABC* genes control the activity of acetyl coenzyme A synthetase. J. Bacteriol.

[b15] Gardner JG, Grundy FJ, Henkin TM, Escalante-Semerena JC (2006). Control of acetyl-coenzyme A synthetase (AcsA) activity by acetylation/deacetylation without NAD+ involvement in *Bacillus subtilis*. J. Bacteriol.

[b16] Glozak MA, Seto E (2007). Histone deacetylases and cancer. Oncogene.

[b17] Gurard-Levin Z, Mrksich M (2008). Combining self-assembled monolayers and mass spectrometry for applications in biochips. Annu. Rev. Anal. Chem.

[b18] Gurard-Levin ZA, Kim J, Mrksich M (2009). Combining mass spectrometry and peptide arrays to profile the specificities of histone deacetylases. ChemBioChem.

[b19] Gurard-Levin ZA, Kilian KA, Kim J, Bahr K, Mrksich M (2010). Peptide arrays identify isoform-selective substrates for profiling endogenous lysine deacetylase activity. ACS Chem. Biol.

[b20] Gurard-Levin ZA, Scholle MD, Eisenberg AH, Mrksich M (2011). High-throughput screening of small molecule libraries using SAMDI mass spectrometry. ACS Comb. Sci.

[b21] Hanahan D (1983). Studies on transformation of *Escherichia coli* with plasmids. J. Mol. Biol.

[b22] Hebert AS, Dittenhafer-Reed KE, Yu W, Bailey DJ, Selen ES, Boersma MD (2013). Calorie restriction and SIRT3 trigger global reprogramming of the mitochondrial protein acetylome. Mol. Cell.

[b23] Herold M, Nierhaus KH (1987). Incorporation of six additional proteins to complete the assembly map of the 50 S subunit from *Escherichia coli* ribosomes. J. Biol. Chem.

[b24] Hildmann C, Riester D, Schwienhorst A (2007). Histone deacetylases: an important class of cellular regulators with a variety of functions. Appl. Microbiol. Biotechnol.

[b25] Hu LI, Lima BP, Wolfe AJ (2010). Bacterial protein acetylation: the dawning of a new age. Mol. Microbiol.

[b26] Hu LI, Chi BK, Kuhn ML, Filippova EV, Walker-Peddakotla AJ, Basell K (2013). Acetylation of the response regulator RcsB controls transcription from a small RNA promoter. J. Bacteriol.

[b27] Huang da W, Sherman BT, Tan Q, Kir J, Liu D, Bryant D (2007). DAVID Bioinformatics Resources: expanded annotation database and novel algorithms to better extract biology from large gene lists. Nucleic Acids Res.

[b28] Jones JD, O'Connor CD (2011). Protein acetylation in prokaryotes. Proteomics.

[b29] Kim GW, Yang XJ (2011). Comprehensive lysine acetylomes emerging from bacteria to humans. Trends Biochem. Sci.

[b30] Kim D, Yu BJ, Kim JA, Lee Y-J, Choi S-G, Kang S (2013). The acetylproteome of Gram-positive model bacterium *Bacillus subtilis*. Proteomics.

[b31] Kitagawa M, Ara T, Arifuzzaman M, Ioka-Nakamichi T, Inamoto E, Toyonaga H (2005). Complete set of ORF clones of *Escherichia coli* ASKA library (a complete set of *E. coli* K-12 ORF archive): unique resources for biological research. DNA Res.

[b32] Klein AH, Shulla A, Reimann SA, Keating DH, Wolfe AJ (2007). The intracellular concentration of acetyl phosphate in *Escherichia coli* Is sufficient for direct phosphorylation of two-component response regulators. J. Bacteriol.

[b33] Kothe U, Wieden HJ, Mohr D, Rodnina MV (2004). Interaction of helix D of elongation factor Tu with helices 4 and 5 of protein L7/12 on the ribosome. J. Mol. Biol.

[b34] Kuhn ML, Majorek KA, Minor W, Anderson WF (2013). Broad-substrate screen as a tool to identify substrates for bacterial Gcn5-related N-acetyltransferases with unknown substrate specificity. Protein Sci.

[b35] Kuhn ML, Zemaitaitis B, Hu LI, Sahu A, Sorensen D, Minasov G (2014). Structural, kinetic and proteomic characterization of acetyl phosphate-dependent bacterial protein acetylation. PLoS One.

[b36] Kumari S, Beatty CM, Browning DF, Busby SJ, Simel EJ, Hovel-Miner G (2000). Regulation of acetyl coenzyme A synthetase in *Escherichia coli*. J. Bacteriol.

[b37] Laptenko O, Lee J, Lomakin I, Borukhov S (2003). Transcript cleavage factors GreA and GreB act as transient catalytic components of RNA polymerase. EMBO J.

[b38] Lee D-W, Kim D, Lee Y-J, Kim J-A, Choi JY, Kang S (2013). Proteomic analysis of acetylation in thermo-philic *Geobacillus kaustophilus*. Proteomics.

[b39] Li R, Gu J, Chen Y-Y, Xiao C-L, Wang L-W, Zhang Z-P (2010). CobB regulates *Escherichia coli* chemotaxis by deacetylating the response regulator CheY. Mol. Microbiol.

[b40] Liang W, Deutscher MP (2012). Post-translational modification of RNase R is regulated by stress-dependent reduction in the acetylating enzyme Pka (YfiQ). RNA.

[b41] Liang W, Malhotra A, Deutscher MP (2011). Acetylation regulates the stability of a bacterial protein: growth stage-dependent modification of RNase R. Mol. Cell.

[b42] Mi H, Muruganujan A, Casagrande JT, Thomas PD (2013). Large-scale gene function analysis with the PANTHER classification system. Nat. Protoc.

[b43] Mrksich M (2008). Mass spectrometry of self-assembled monolayers: a new tool for molecular surface science. ACS Nano.

[b44] Nickels BE, Hochschild A (2004). Regulation of RNA polymerase through the secondary channel. Cell.

[b45] Papanastasiou M, Orfanoudaki G, Koukaki M, Kountourakis N, Sardis MF, Aivaliotis M (2013). The *Escherichia coli* peripheral inner membrane proteome. Mol. Cell Proteomics.

[b46] Rardin MJ, Newman JC, Held JM, Cusack MP, Sorensen DJ, Li B (2013). Label-free quantitative proteomics of the lysine acetylome in mitochondria identifies substrates of SIRT3 in metabolic pathways. Proc. Natl. Acad. Sci. USA.

[b47] Rutherford ST, Lemke JJ, Vrentas CE, Gaal T, Ross W, Gourse RL (2007). Effects of DksA, GreA, and GreB on transcription initiation: insights into the mechanisms of factors that bind in the secondary channel of RNA polymerase. J. Mol. Biol.

[b48] Sanville MC, Stols L, Quartey P, Kim Y, Dementieva I, Donnelly M (2003). A less laborious approach to the high-throughput production of recombinant proteins in *Escherichia coli* using 2-liter plastic bottles. Protein Expr. Purif.

[b49] Schilling B, Rardin M, Hunter C, Zawadzka A, Danielson SR, Cusack M (2012).

[b50] Shajani Z, Sykes MT, Williamson JR (2011). Assembly of bacterial ribosomes. Annu. Rev. Biochem.

[b51] Silhavy TJ, Berman ML, Enquist LW (1984).

[b52] Sol EM, Wagner SA, Weinert BT, Kumar A, Kim HS, Deng CX (2012). Proteomic investigations of lysine acetylation identify diverse substrates of mitochondrial deacetylase sirt3. PLoS One.

[b53] Jörg Soppa, “Protein Acetylation in Archaea, Bacteria, and Eukaryotes” (2010). 10.1155/2010/820681.

[b54] Starai VJ, Escalante-Semerena JC (2004). Identification of the protein acetyltransferase (Pat) enzyme that acetylates acetyl-CoA synthetase in *Salmonella enterica*. J. Mol. Biol.

[b55] Starai VJ, Celic I, Cole RN, Boeke JD, Escalante-Semerena JC (2002). Sir2-dependent activation of acetyl-CoA synthetase by deacetylation of active lysine. Science.

[b56] Thao S, Escalante-Semerena JC (2011). Control of protein function by reversible N[epsilon]-lysine acetylation in bacteria. Curr. Opin. Microbiol.

[b57] Thao S, Chen C, Zhu H, Escalante-Semerena J (2010). N(epsilon)-lysine acetylation of a bacterial transcription factor inhibits Its DNA-binding activity. PLoS One.

[b58] Tucker AC, Escalante-Semerena JC (2010). Biologically active isoforms of CobB sirtuin deacetylase in *Salmonella enterica* and *Erwinia amylovora*. J. Bacteriol.

[b59] Wagner G, Payne R (2013). Widespread and enzyme-independent N{epsilon}-acetylation and N{epsilon}-succinylation in the chemical conditions of the mitochondrial matrix. J. Biol. Chem.

[b60] Wang Q, Zhang Y, Yang C, Xiong H, Lin Y, Yao J (2010). Acetylation of metabolic enzymes coordinates carbon source utilization and metabolic flux. Science.

[b61] Weinert BT, Iesmantavicius V, Wagner SA, Scholz C, Gummesson B, Beli P (2013). Acetyl-phosphate is a critical determinant of lysine acetylation in *E. coli*. Mol. Cell.

[b62] Wolfe AJ (2005). The acetate switch. Microbiol. Mol. Biol. Rev.

[b63] Wolfe AJ, Chang D-E, Walker JD, Seitz-Partridge JE, Vidaurri MD, Lange CF (2003). Evidence that acetyl phosphate functions as a global signal during biofilm development. Mol. Microbiol.

[b64] Wu X, Vellaichamy A, Wang D, Zamdborg L, Kelleher NL, Huber SC (2013). Differential lysine acetylation profiles of *Erwinia amylovora* strains revealed by proteomics. J. Proteomics.

[b65] Yang X-J, Seto E (2008a). Lysine acetylation: codified crosstalk with other posttranslational modifications. Mol. Cell.

[b66] Yang X-J, Seto E (2008b). The Rpd3/Hda1 family of lysine deacetylases: from bacteria and yeast to mice and men. Nat. Rev. Mol. Cell Biol.

[b67] Zhang J, Sprung R, Pei J, Tan X, Kim S, Zhu H (2009). Lysine acetylation is a highly abundant and evolutionarily conserved modification in *Escherichia coli*. Mol. Cell Proteomics.

[b68] Zhang K, Zheng S, Yang JS, Chen Y, Cheng Z (2013). Comprehensive profiling of protein lysine acetylation in *Escherichia coli*. J. Proteome Res.

[b69] Zhao K, Chai X, Marmorstein R (2004). Structure and substrate binding properties of CobB, a Sir2 homolog protein deacetylase from *Escherichia coli*. J. Mol. Biol.

