# Supplementary material for: The E. coli sirtuin CobB shows no preference for enzymatic and nonenzymatic lysine acetylation substrate sites
Source: Microbiologyopen. 2014 Nov 22;4(1):66–83. doi: 10.1002/mbo3.223 (PMC4335977; doi:10.1002/mbo3.223)
Supplement: Supplementary file 1 [file mbo30004-0066-sd1.pdf]

Supplemental Figure Legends

Figure S1. Ontology assessment of *cobB*-sensitive acetyllysine sites

51 proteins that exhibited significant up-regulation of 69 acetyllysine sites in the *cobB* mutant relative to WT were subjected to ontology analysis using Panther. (**A**) Molecular function GO categories, and (**B**) Biological processes GO categories.

Figure S2. Anti-acetyllysine Western immunoblot analyses. (**A**) *E. coli* WT (strain MG1655) and isogenic mutants *cobB* (strain AJW5037), *yfiQ* (strain AJW5184) and *cobB yfiQ* (strain AJW5118) were aerated at 37°C in TB7 and harvested at 3 time points, when the OD_{610} reached 0.5 or 1.0, and then at 8 hours. The white arrow points to a 70-kDa band that depends on YfiQ and CobB. (**B**) WT (strain AJW678), the *cobB* mutant (strain AJW4343), the *yfiQ* mutant (strain AJW4344) and the double *cobB yfiQ* mutant (strain AJW5109) were aerated in TB7 at 37°C. and harvested at 3 time points, when the OD_{610} reached 0.5 or 1.0, and then at 8 hours. The white arrow points to a 70-kDa band that depends on YfiQ and CobB.

Figure S1

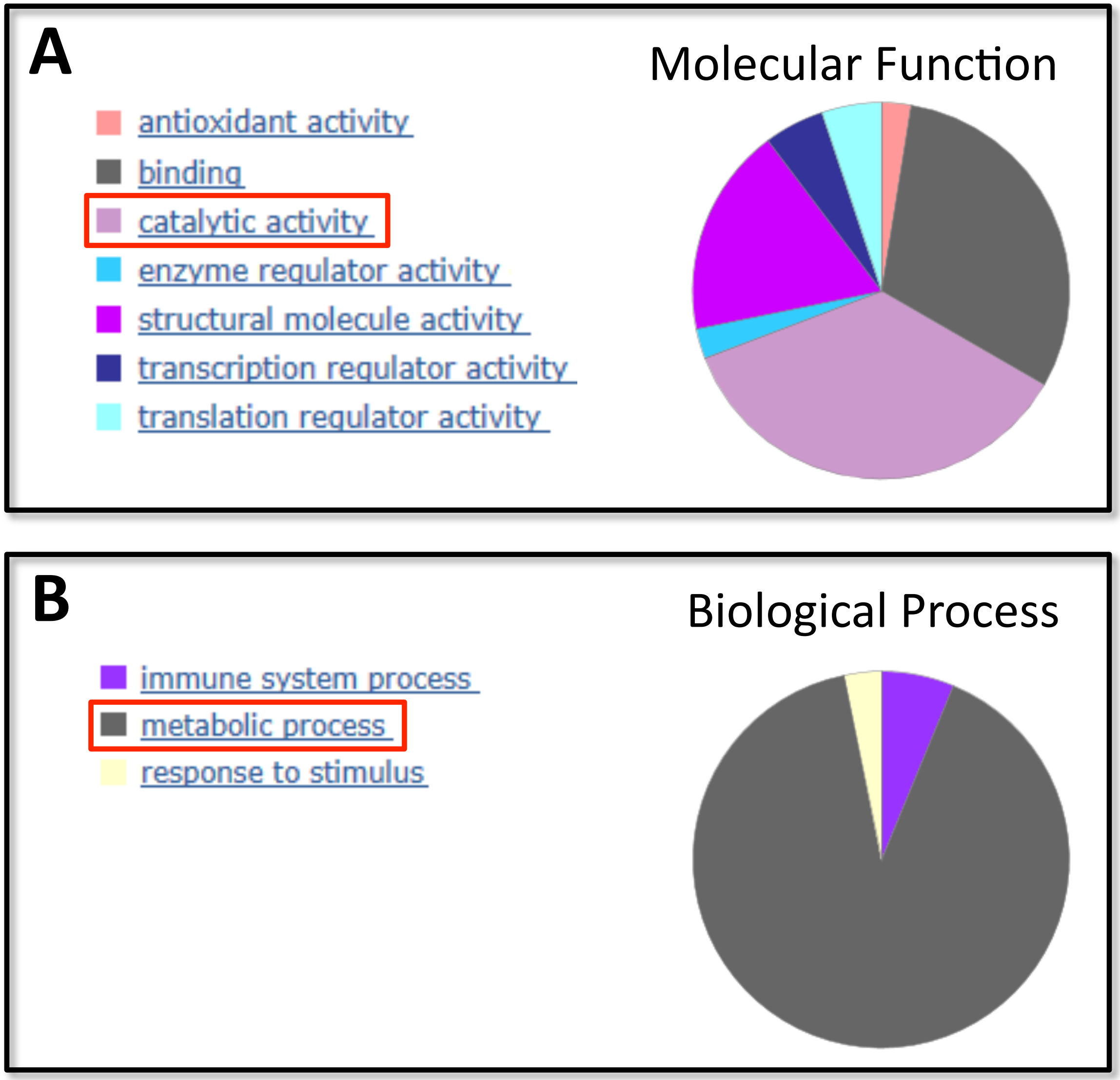
A
Molecular Function
antioxidant activity
binding
catalytic activity
enzyme regulator activity
structural molecule activity
transcription regulator activity
translation regulator activity
B
Biological Process
immune system process
metabolic process
response to stimulus

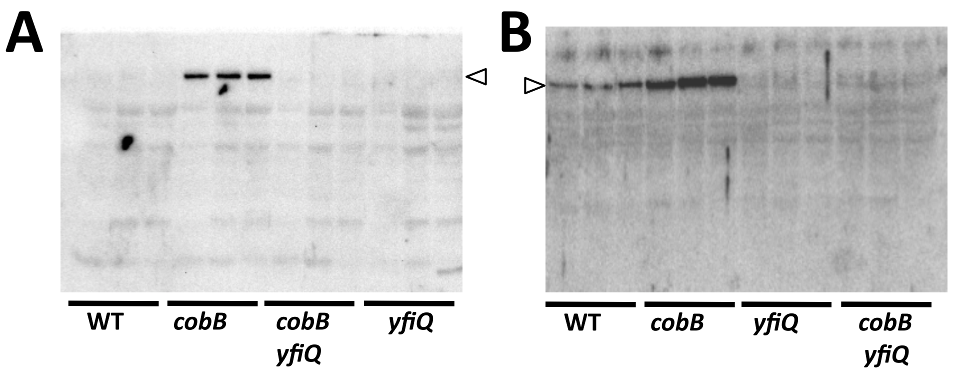

Figure S2

Supplemental Table Legends

Table S1. Acetyl sites that are significantly regulated in the *cobB* mutant relative to WT. Defined as ratio >2 with P value <0.05 in at least 3 out of 4 biological replicates.

Table S2. Ontology analysis of proteins with CobB regulated acetyllysines. (**A**) Panther Ontology analysis, (**B**) Pathway enrichment analysis, KEGG pathways, and (**C**) Pathway enrichment analysis, Gene Ontology.

Table S3. Analysis of acetylated ribosomal proteins. (**A**) Ribosomal proteins with acetylated peptides that were significantly upregulated in the *cobB* mutant relative to the WT mutant. (**B**) Ribosomal proteins with acetylated peptides that were significantly upregulated in the *cobB* mutant relative to the WT parent (top) or significantly upregulated in the *ackA* mutant relative to the WT parent (bottom). (**C**) Acetyllysines that were upregulated in either the *cobB* mutant, the *ackA* mutant or both.

Table S4. The average deacetylase activity of recombinant CobB and lysates (WT and *cobB* deletion mutant) towards an acetylated peptide library using the SAMDI technique.

Table S5. Analyzing the residues neighboring CobB-sensitive and not sensitive lysines. 69 sites that exhibited significantly upregulated acetylation (ratio >2 with a P value < 0.05 in at least 3 out of 4 biological replicates) in the *cobB* mutant relative to WT were analyzed. The amino acid frequencies were determined for the 51 proteins that had significantly upregulated acetyllysine modifications. The frequencies of

the residues in the -1 and +1 positions relative to both the 69 CobB-sensitive and 760 non-CobB-sensitive lysines were determined. The frequencies of each amino acid neighboring these lysines were normalized to the frequencies of each amino acid in the 51 proteins. 10 lysine residues that were the terminal amino acid of a protein were not included in this analysis due to these residues lacking a +1 position.

Table S6. Protein structures from the Protein Data Bank (PDB) used for 3D analysis of CobB substrate proteins. Substrate lysines for each protein identified by quantitative mass spectrometry are shown, as well as their corresponding lysine numbering in the structure. Homologous structures were used if no structure for *E. coli* had been determined. The type of secondary structure that contains the substrate lysine is also indicated.

Table S7. Amino acid residues adjacent to CobB substrate lysines in 3D. For candidate CobB substrates, amino acid residues that are adjacent to the substrate acetyllysine in 3D are shown. When appropriate, the residues from homologous structures are shown, but the corresponding residues in *E. coli* were used for further analysis. Yellow highlight indicates the substrate lysine residue, while cyan highlights the adjacent residues identified from 3D structures. LuxS from *Bacillus* has an insertion prior to the substrate acetyllysine that is not present in *E. coli*. Therefore, the corresponding adjacent residue in this region of the protein is not clear. If the identity of the residue adjacent to the acetyllysine was not clear from the structure, that residue is indicated with "X."

Table S8. Correlation of adjacent residues to the CobB substrate acetyllysine in 1D versus in 3D. To determine the correlation, the three amino acids in 3D were

compared with the SAMDI deacetylation profile for recombinant CobB (**Fig. 3**). An "X" indicates that the residue adjacent to the acetyllysine was unclear. A question mark indicates those 3D sequences whose correlations could not be determined due to the absence of an adjacent residue.

Table S9. Proteins with acetylated lysines found in the *cobB ackA* mutant and identified by mass spectrometry. A gel slice corresponding to the band indicated by the upper black arrow in Figure 5B was cut from a parallel SDS-polyacrylamide gel and subjected to mass spectrometry: (A) EF-Tu (TufA), (B) Pgk, (C) FbaA, and (D) GapA.
