# Supplementary material for: The E. coli sirtuin CobB shows no preference for enzymatic and nonenzymatic lysine acetylation substrate sites
Source: Microbiologyopen. 2014 Nov 22;4(1):66–83. doi: 10.1002/mbo3.223 (PMC4335977; doi:10.1002/mbo3.223)
Supplement: Supplementary file 7 [file mbo30004-0066-sd7.docx]

**Table S6.** Protein structures from the Protein Data Bank (PDB) used for 3D analysis of CobB substrate proteins. Substrate lysines for each protein identified by quantitative mass spectrometry are shown, as well as their corresponding lysine numbering in the structure. Homologous structures were used if no structure for *E. coli* had been determined. The type of secondary structure that contains the substrate lysine is also indicated.

| *E. coli* Gene | PDB ID | Chain | Organism | Acetyl Site  (*E. coli)* | Lysine # in Structure | Secondary Structure |
| --- | --- | --- | --- | --- | --- | --- |
| *accB* | 4HR7 | B | *Escherichia coli* | 100 | 100 | loop |
| *aceE* | 2IEA | A | *Escherichia coli* | 404 | 404 | loop-disordered |
| *acpP* | 2K93 | A | *Escherichia coli* | 10 | 9 | alpha-helix |
| *bcp* | 3GKK | A | *Xanthomonas campestris* | 114 | 116 | loop |
| *crr* | 1F3G | A | *Escherichia coli* | 6 | 6 | loop-disordered |
| *cspE* | 3I2Z | A | *Salmonella typhimurium* | 9 | 9 | beta-strand |
| *csrA* | 1Y00 | A | *Escherichia coli* | 55 | 55 | loop |
| *dnaG* | 1DDE | A | *Escherichia coli* | 229 | 229 | loop |
| *dnaK* | 2KHO | A | *Escherichia coli* | 514 | 514 | alpha-helix |
| *frr* | 1EK8 | A | *Escherichia coli* | 80 | 80 | alpha-helix |
| *gadA* | 1XEY | A | *Escherichia coli* | 4 | 4 | alpha-helix |
| *gpmA* | 1E58 | A | *Escherichia coli* | 113 | 112 | alpha-helix |
| *greA* | 1GRJ | A | *Escherichia coli* | 63 | 63 | alpha-helix |
| *greA* | 1GRJ | A | *Escherichia coli* | 43 | 43 | alpha-helix end |
| *groS* | 1PCQ | U | *Escherichia coli* | 74 | 74 | loop |
| *grxA* | 1QFN | A | *Escherichia coli* | 45 | 45 | alpha-helix |
| *guaB* | 3TSD | B | *Bacillus anthracis* | 203 | 206 | alpha-helix |
| *hns* | 3NR7 | A | *Salmonella typhimurium* | 6 | 6 | alpha-helix |
| *hns* | 2L93 | A | *Salmonella enterica* | 96 | 96 | loop |
| *hns* | 3NR7 | A | *Salmonella typhimurium* | 83 | 83 | loop-disordered |
| *hupA* | 1MUL | A | *Escherichia coli* | 83 | 83 | alpha-helix end |
| *hupA* | 1MUL | A | *Escherichia coli* | 18 | 18 | alpha-helix end |
| *hupA* | 1MUL | A | *Escherichia coli* | 67 | 67 | loop-disordered |
| *hupA* | 1MUL | A | *Escherichia coli* | 70 | 70 | loop-disordered |
| *hupB* | 2O97 | B | *Escherichia coli* | 67 | 67 | loop-disordered |
| *infA* | 1AH9 | A | *Escherichia coli* | 64 | 63 | beta-strand |
| *infB* | 1ND9 | A | *Escherichia coli* | 58 | 58 | loop-disordered |
| *luxS* | 1YCL | A | *Bacillus subtilis* | 163 | 149 | alpha-helix |
| *pdxH* | 1DNL | A | *Escherichia coli* | 159 | 159 | alpha-helix |
| *phnA* | 2AKL | A | *Pseudomonas aeruginosa* | 94 | 97 | beta-strand |
| *phnA* | 2AKL | A | *Pseudomonas aeruginosa* | 104 | 107 | loop |
| *pyrG* | 2AD5 | A | *Escherichia coli* | 432 | 432 | loop-disordered |
| *rcsB* | 1P4W | A | *Erwinia amylovora* | 154 | 154 | alpha-helix end |
| *rimI* | 2CNS | A | *Salmonella typhimurium* | 30 | 30 | alpha-helix end |
| *rplK* | 3J37 | K | *Escherichia coli* | 81 | 81 | alpha-helix end |
| *rplL* | 1CTF | A | *Escherichia coli* | 71 | 70 | alpha-helix |
| *rplL* | 1CTF | A | *Escherichia coli* | 82 | 81 | alpha-helix end |
| *rplQ* | 3J37 | R | *Escherichia coli* | 35 | 35 | beta-strand |
| *rpmC* | 3J37 | 2 | *Escherichia coli* | 44 | 44 | alpha-helix |
| *rpmE* | 3J37 | 4 | *Escherichia coli* | 8 | 8 | loop |
| *rpmG* | 3J37 | 6 | *Escherichia coli* | 50 | 49 | beta-strand |
| *rpmG* | 3J37 | 6 | *Escherichia coli* | 10 | 9 | beta-strand |
| *rpmG* | 3J37 | 6 | *Escherichia coli* | 33 | 32 | loop |
| *rpsJ* | 1VS5 | J | *Escherichia coli* | 59 | 59 | loop |
| *rpsQ* | 1VS5 | Q | *Escherichia coli* | 71 | 70 | loop |
| *rpsU* | 1VS5 | U | *Escherichia coli* | 25 | 24 | alpha-helix end |
| *tig* | 1W26 | A | *Escherichia coli* | 327 | 327 | loop |
| *tig* | 1W26 | A | *Escherichia coli* | 361 | 361 | loop |
| *tnaA* | 2C44 | A | *Escherichia coli* | 459 | 459 | loop |
| *tsf* | 1EFU | D | *Escherichia coli* | 214 | 213 | alpha-helix |
| *tsf* | 1EFU | D | *Escherichia coli* | 222 | 221 | alpha-helix |
| *tuf1* | 1EFU | A | *Escherichia coli* | 38 | 37 | alpha-helix end |
| *tuf1* | 1EFU | A | *Escherichia coli* | 57 | 56 | loop-disordered |
| *yejL* | 2JRX | A | *Escherichia coli* | 73 | 73 | loop |
| *yihD* | 2KO6 | A | *Shigella flexneri* | 70 | 70 | loop |
| *yjbJ* | 1RYK | A | *Escherichia coli* | 11 | 11 | alpha-helix |
| *yjbJ* | 1RYK | A | *Escherichia coli* | 3 | 3 | loop |
