# Supplementary material for: The E. coli sirtuin CobB shows no preference for enzymatic and nonenzymatic lysine acetylation substrate sites
Source: Microbiologyopen. 2014 Nov 22;4(1):66–83. doi: 10.1002/mbo3.223 (PMC4335977; doi:10.1002/mbo3.223)
Supplement: Supplementary file 8 [file mbo30004-0066-sd8.docx]

**Table S7.** For candidate CobB substrates, amino acid residues that are adjacent to the substrate acetyl-lysine in 3D are shown. When appropriate, the residues from homologous structures are shown, but the corresponding residues in *E. coli* were used for further analysis. Yellow highlight indicates the substrate lysine residue, while cyan highlights the adjacent residues identified from 3D structures. LuxS from *Bacillus* has an insertion prior to the substrate acetyl-lysine that is not present in *E. coli.* Therefore, the corresponding adjacent residue in this region of the protein is not clear. If the identity of the residue adjacent to the acetyl-lysine was not clear from the structure, that residue is indicated with “X.”

| ***E. coli* Gene** |  |  | **Protein Sequence from Structure** | | | | | | | | |  |  | **Sequence in 3D from Structure** | ***E. coli* Sequence in 3D** | ***E. coli* Primary Sequence** |
| --- | --- | --- | --- | --- | --- | --- | --- | --- | --- | --- | --- | --- | --- | --- | --- | --- |
| *accB* |  |  | S | P | D | **A** | **K** | **A** | F | I | E |  |  | AKA | AKA | AKA |
| *acpP* |  |  | **E** | R | V | K | **K** | I | I | G | **Q** |  |  | EKQ | EKQ | KKI |
| *bcp* |  |  | N | M | Y | **G** | **K** | Q | **V** | L | G |  |  | GKV | GKY | GKT |
| *cspE* |  |  | K | G | **N** | V | **K** | **W** | F | N | E |  |  | NKW | NKW | VKW |
| *csrA* |  |  | I | Q | A | **E** | **K** | **S** | Q | Q | S |  |  | EKS | EKS | EKS |
| *dnaG* |  |  | N | D | T | **P** | **K** | **Y** | L | N | S |  |  | PKY | PKY | PKY |
| *dnaK* |  |  | **D** | E | I | Q | **K** | M | V | R | **D** |  |  | DKD | DKD | QKM |
| *frr* |  |  | **P** | A | V | E | **K** | A | I | M | **A** |  |  | PKA | PKA | EKA |
| *gadA* |  |  |  | M | D | **Q** | **K** | **L** | L | T | D |  |  | QKL | QKL | QKL |
| *gpmA* |  |  | **D** | E | Q | V | **K** | Q | W | R | **R** |  |  | DKR | DKR | VKQ |
| *greA* |  |  | **E** | G | R | I | **K** | D | I | E | **A** |  |  | EKA | EKA | IKD |
| *greA* |  |  | H | G | D | L | **K** | **E** | N | A | E |  |  | XKE | XKE | LKE |
| *groS* |  |  | G | Y | **G** | V | **K** | S | **E** | K | I |  |  | GKE | GKE | VKS |
| *grxA* |  |  | E | G | I | T | **K** | E | D | L | **Q** |  |  | XKQ | XKQ | TKE |
| *guaB* |  |  | **K** | D | I | E | **K** | V | I | E | **F** |  |  | KKF | KKK | GKA |
| *hns* |  |  | **S** | E | A | L | **K** | I | L | N | **N** |  |  | SKN | SKN | LKI |
| *hns* |  |  | A | R | P | **A** | **K** | **Y** | S | Y | V |  |  | AKY | AKY | AKY |
| *hupA* |  |  | F | V | S | **G** | **K** | A | L | K | **D** |  |  | GKD | GKD | GKA |
| *hupA* |  |  | A | E | L | S | **K** | T | Q | A | **K** |  |  | XKK | XKK | SKT |
| *infA* |  |  | Y | D | L | **S** | **K** | **G** | R | I | V |  |  | SKG | SKG | SKG |
| *luxS* |  |  | L | S | Q | D | **K** | E | E | L | **L** |  |  | XKL | XKQ | PKE |
| *pdxH* |  |  | **I** | L | E | S | **K** | F | L | E | **L** |  |  | IKL | IKL | SKF |
| *phnA* |  |  | D | I | **D** | C | **K** | I | **D** | G | I |  |  | DKD | DKD | CKI |
| *phnA* |  |  | A | M | **K** | L | **K** | S | **E** | F | V |  |  | KKE | KKE | LKS |
| *rcsB* |  |  | R | L | S | P | **K** | E | S | E | **V** |  |  | XKV | XKV | PKE |
| *rimI* |  |  | P | W | S | E | **K** | T | F | F | **G** |  |  | XKG | XKS | EKT |
| *rplK* |  |  | **V** | L | L | K | **K** | A | A | G | I |  |  | VKX | VKX | KKA |
| *rplL* |  |  | **V** | A | V | I | **K** | A | V | R | **G** |  |  | VKG | VKG | IKA |
| *rplL* |  |  | G | L | G | L | **K** | E | A | K | **D** |  |  | XKD | XKD | LKE |
| *rplQ* |  |  | H | E | **I** | I | **K** | T | **T** | L | P |  |  | IKT | IKT | IKT |
| *rpmC* |  |  | **S** | H | L | L | **K** | Q | V | R | **R** |  |  | SKR | SKR | LKQ |
| *rpmE* |  |  | D | I | H | **P** | **K** | **Y** | E | E | I |  |  | PKY | PKY | PKY |
| *rpmG* |  |  | H | V | **I** | Y | **K** | **E** | A | K | I |  |  | IKE | IKE | YKE |
| *rpmG* |  |  | R | E | **K** | I | **K** | L | **V** | S | S |  |  | KKV | KKV | IKL |
| *rpmG* |  |  | T | K | P | **E** | **K** | **L** | E | L | K |  |  | EKL | EKL | EKL |
| *rpsJ* |  |  | P | H | V | **N** | **K** | **D** | A | R | D |  |  | NKD | NKD | NKD |
| *rpsQ* |  |  | L | S | K | **T** | **K** | **S** | W | T | L |  |  | TKS | TKS | TKS |
| *rpsU* |  |  | R | S | C | **E** | **K** | **A** | G | V | L |  |  | EKA | EKA | EKA |
| *tig* |  |  | G | G | N | **E** | **K** | **Q** | A | L | E |  |  | EKQ | EKQ | EKQ |
| *tig* |  |  | T | N | E | **L** | **K** | **A** | D | E | E |  |  | LKA | LKA | LKA |
| *tnaA* |  |  | T | Y | E | **P** | **K** | **V** | L | R | H |  |  | PKV | PKV | PKV |
| *tsf* |  |  | **E** | I | A | E | **K** | M | V | E | **G** |  |  | EKG | EKG | EKM |
| *tsf* |  |  | **G** | R | M | K | **K** | F | T | **G** | E |  |  | GKG | GKG | KKF |
| *tuf1* |  |  | **T** | V | L | A | **K** | T | Y | G | G |  |  | TKX | TKX | AKT |
| *yejL* |  |  | I | N | E | **D** | **K** | **A** | H | L | E |  |  | DKA | DKA | DKA |
| *yihD* |  |  | P | G | L | **Q** | **K** | **D** | Y | E | E |  |  | QKD | QKD | QKD |
| *yjbJ* |  |  | G | **G** | N | W | **K** | Q | F | K | **G** |  |  | GKG | GKG | WKQ |
| *yjbJ* |  |  |  |  | M | **N** | **K** | **D** | E | A | G |  |  | NKD | NKD | NKD |
