# Supplementary material for: The E. coli sirtuin CobB shows no preference for enzymatic and nonenzymatic lysine acetylation substrate sites
Source: Microbiologyopen. 2014 Nov 22;4(1):66–83. doi: 10.1002/mbo3.223 (PMC4335977; doi:10.1002/mbo3.223)
Supplement: Supplementary file 9 [file mbo30004-0066-sd9.docx]

**Table S8.** Correlation of adjacent residues to the CobB substrate acetyllysine in 1D versus in 3D. To determine the correlation, the three amino acids in 3D were compared with the SAMDI deacetylation profile for recombinant CobB (**Fig. 3**). An “X” indicates that the residue adjacent to the acetyllysine was unclear. A question mark indicates those 3D sequences whose correlations could not be determined due to the absence of an adjacent residue.

| *E. coli* Gene | *E. coli* Sequence in 3D | Correlate with SAMDI? | Secondary structure |
| --- | --- | --- | --- |
| *accB* | AKA | YES | loop |
| *acpP* | EKQ | NO | alpha-helix |
| *bcp* | GKY | YES | loop |
| *cspE* | NKW | YES | beta-strand |
| *csrA* | EKS | NO | loop |
| *dnaG* | PKY | YES | loop |
| *dnaK* | DKD | NO | alpha-helix |
| *frr* | PKA | NO | alpha-helix |
| *gadA* | QKL | YES | alpha-helix |
| *gpmA* | DKR | NO | alpha-helix |
| *greA* | EKA | NO | alpha-helix |
| *groS* | GKE | YES | loop |
| *grxA* | XKQ | ? | alpha-helix |
| *guaB* | KKK | NO | alpha-helix |
| *hns* | SKN | NO | alpha-helix |
| *hns* | AKY | YES | loop |
| *infA* | SKG | NO | beta-strand |
| *luxS* | XKQ | ? | alpha-helix |
| *pdxH* | IKL | YES | alpha-helix |
| *phnA* | DKD | NO | beta-strand |
| *phnA* | KKE | NO | loop |
| *rplL* | VKG | YES | alpha-helix |
| *rplQ* | IKT | YES | beta-strand |
| *rpmC* | SKR | YES | alpha-helix |
| *rpmE* | PKY | YES | loop |
| *rpmG* | IKE | YES | beta-strand |
| *rpmG* | KKV | YES | beta-strand |
| *rpmG* | EKL | YES | loop |
| *rpsJ* | NKD | YES | loop |
| *rpsQ* | TKS | YES | loop |
| *tig* | EKQ | NO | loop |
| *tig* | LKA | YES | loop |
| *tnaA* | PKV | YES | loop |
| *tsf* | EKG | YES | alpha-helix |
| *tsf* | GKG | NO | alpha-helix |
| *yejL* | DKA | NO | loop |
| *yihD* | QKD | NO | loop |
| *yjbJ* | GKG | NO | alpha-helix |
| *yjbJ* | NKD | NO | loop |
